# Multistage Delivery Nanoparticle Facilitates Efficient CRISPR/dCas9 Activation and Tumor Growth Suppression In Vivo

**DOI:** 10.1002/advs.201801423

**Published:** 2018-10-25

**Authors:** Qi Liu, Kai Zhao, Chun Wang, Zhanzhan Zhang, Chunxiong Zheng, Yu Zhao, Yadan Zheng, Chaoyong Liu, Yingli An, Linqi Shi, Chunsheng Kang, Yang Liu

**Affiliations:** ^1^ State Key Laboratory of Medicinal Chemical Biology Key Laboratory of Functional Polymer Materials of Ministry of Education College of Chemistry Nankai University Tianjin 300071 China; ^2^ Tianjin Neurological Institute Key Laboratory of Post‐neurotrauma Neuro‐repair and Regeneration in Central Nervous System Ministry of Education and Tianjin City Department of Neurosurgery Tianjin Medical University General Hospital Tianjin 300052 China

**Keywords:** cancer therapy, CRISPR/dCas9, gene regulation, miR‐524, multistage delivery

## Abstract

CRISPR/dCas9 systems can precisely control endogenous gene expression without interrupting host genomic sequence and have provided a novel and feasible strategy for the treatment of cancers at the transcriptional level. However, development of CRISPR/dCas9‐based anti‐cancer therapeutics remains challenging due to the conflicting requirements for the design of the delivery system: a cationic and membrane‐binding surface facilitates the tumor accumulation and cellular uptake of the CRISPR/dCas9 system, but hinders the circulating stability in vivo. Here, a multistage delivery nanoparticle (MDNP) that can achieve tumor‐targeted delivery of CRISPR/dCas9 systems and restore endogenous microRNA (miRNA) expression in vivo is described. MDNP is designed as a core‐shell structure in which the shell is made of a responsive polymer that endows MDNP with the capability to present different surface properties in response to its surrounding microenvironment, allowing the MNDP overcoming multiple physiological barriers and delivering the payload to tumor tissues with an optimal efficiency. Systemic administration of MDNP/dCas9‐miR‐524 to tumor‐bearing mice achieved effective upregulation of miR‐524 in tumors, leading to the simultaneous interferences of multiple signal pathways related to cancer cell proliferation and presenting remarkable tumor growth retardation, suggesting the feasibility of utilizing MDNP to achieve tumor‐targeting delivery of CRISPR/dCas9 with sufficient levels to realize its therapeutic effects.

## 1 Introduction

Clustered regularly interspaced short palindromic repeat (CRISPR)/CRISPR‐associated protein 9 (Cas9) system has emerged as a robust and versatile genome‐editing platform.^1^ By fusing the nuclease‐inactivated Cas9 (dCas9) with transcription activators and repressors, CRISPR/dCas9 system can achieve precise and efficient control of gene expression without cutting the target DNA, because the dCas9 can be directed to virtually anywhere in the genome using a short guide RNA (sgRNA).^2^ Since suppression of oncogenes or upregulation of tumor suppressor genes has been proved to be effective avenue to retard growth of tumors,^3^ therapeutics based on CRISPR/dCas9 have tremendous potential for curing cancers at transcription level.^4^ Compared to CRISPR/Cas9 systems that involve cutting and mutating the genome, CRISPR/dCas9 systems can regulate multiple oncogenic targets synergistically via modulating the transcription of endogenous microRNAs (miRNAs),^2^ providing a safer and more natural way to treat cancers.^5^ Despite several successful demonstration of applying CRISPR/dCas9 in cell line–based experiments,^2^, ^6^ in vivo gene transcriptional regulation based on CRISPR/dCas9 for cancer therapy remains challenging due to the poor transport of CRISPR/dCas9 across multiple physiological barriers to cancer cells.

The intrinsic mechanism of CRISPR system determines that it can only function inside its target cells.^2^ To this end, most of CRISPR/dCas9 studies so far have achieved the delivery via viral vectors.^7^ However, high immunogenicity and safety concerns limit the clinical potential of viral vector–based CRISPR gene therapies.^8^ Difficulties in the clinical translation of viral vectors empowered the investigations on synthetic vectors to accomplish such delivery.^9^ To date, several strategies based on cationic liposome,^10^ cationic polymer nanoparticles,^10^ and gold nanoparticles were successfully developed for delivery of CRISPR systems.^11^ However, all these nonviral delivery strategies have been designed and optimized for efficient CRISPR/Cas9‐based gene editing in vitro and in vivo. Although some of these delivery strategies might be able to adapt to CRISPR/dCas9, a specifically designed delivery strategy for CRISPR/dCas9 is clearly anticipated to realize its full potential especially in cancer gene therapy.

**Figure 1:**
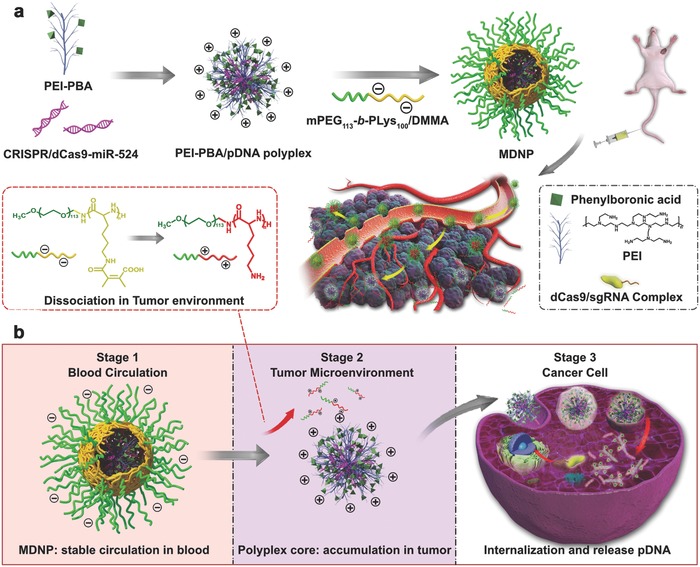
a) Schematic illustration for the preparation of MDNP and delivery process after intravenous injection. b) Multistage delivery of CRISPR/dCas9 system from blood circulation to tumor cells via MDNP. Stage 1: MDNP maintains stable during circulating in bloodstream. Stage 2: the dissociation of the polymer coating and exposure of the cationic core lead to the accumulation of the polyplex in tumor tissues. Stage 3: the internalization by cancer cells and the release of CRISPR/dCas9 system enable the regulation of the gene expression.

From a delivery perspective, a therapeutic agent has to undergo three consecutive stages in a successful CRISPR/dCas9‐based cancer treatment. Stage 1: the agent maintains stable during circulating in bloodstream, Stage 2: the agent accumulates in tumor tissues, and Stage 3: the agent enters into cancer cells, escapes from endosomes/lysosomes to cytoplasm, and enters nucleus to regulate the expression of the target gene.^12^ However, these stages require the delivery system to have completely different surface properties, it is therefore challenging to integrate these three capabilities on a single delivery system. Herein, we present a nanoparticle‐based delivery system that can achieve multistage delivery of CRISPR/dCas9 system in vivo via intravenous administration and induce the transcriptional activation of the tumor suppressor gene miR‐524 in cancer cells. The multistage delivery nanoparticle (denoted as MDNP) has a core–shell structure, in which the core is a cationic polyplex made from CRISPR/dCas9 plasmid DNA (pDNA) and phenylboronic acid (PBA)‐modified low molecular weight polyethyleneimine (PEI–PBA), whereas the shell is formed by 2,3‐dimethylmaleic anhydride (DMMA)‐modified poly(ethylene glycol)‐*b*‐polylysine (mPEG_113_‐*b*‐PLys_100_/DMMA) (**Figure** **1**a). Aiming to overcome the multiple physiological barriers in the delivery of CRISPR/dCas9 system from blood to tumor cells, MDNP is designed to exhibit corresponding surface properties at different delivery stages. When circulating in bloodstream, MDNP maintains the core–shell structure. The polymer shell endows MDNP with negatively charged, PEGylated surface that effectively reduces the immune clearance.^13^ As entering tumor tissues, the acidic microenvironment induces the decomposition of DMMA groups in the polymer shell of MDNP,^14^ leading to the rapid conversion of mPEG_113_‐*b*‐PLys_100_/DMMA from an anionic polymer to a cationic polymer (mPEG_113_‐*b*‐PLys_100_). Consequently, the polymer shell is detached from the MDNP core due to electrostatic repulsion, leading to exposure of the polyplex core with a cationic surface, which enhances the tumor accumulation and cell internalization.^15^ Moreover, since cancer cells usually have high levels of surface sialylation, the PBA groups on the polyplex can bind with those sialic acid and eventually enhance the internalization of the polyplex into cancer cells.^16^ After internalization, the PEI in the polyplex triggers the endosomal disruption and the release of CRISPR/dCas9 pDNA into the cytoplasm due to the proton sponge effect and the less entanglement between the pDNA and low molecular weight PEI–PBA,^17^ respectively (Figure 1b). With this multistage delivery strategy, MDNP achieved the efficient delivery of CRISPR/dCas9–miR‐524 system and successfully inhibited the tumor growth in mice, providing a feasible approach for the development of CRISPR‐based cancer gene therapeutics.

## 2 Results

### 2.1 Design, Synthesis, and Analysis of MDNP

**Figure 2:**
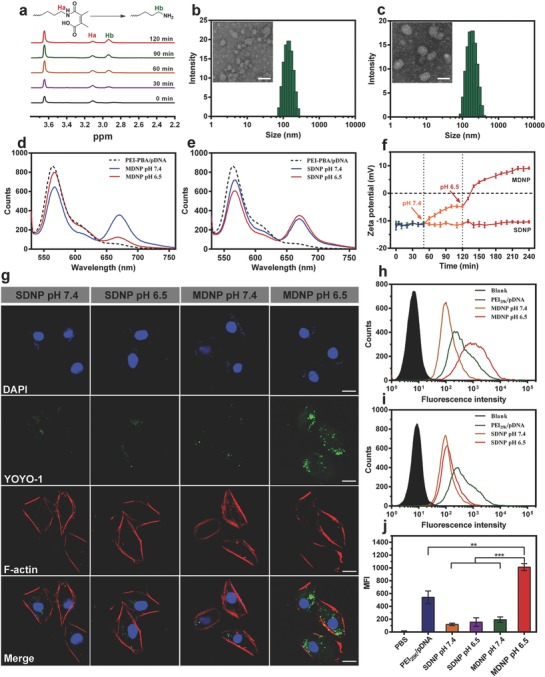
a) ^1^H NMR spectra of mPEG_113_‐*b*‐PLys_100_/DMMA after being incubated at pH 6.5 in D_2_O/DCl (25 °C) for different time periods (Ha and Hb are attributed to the methylene protons adjacent to the amino group and amide bond, respectively). b,c) TEM images and DLS measurements of the CRISPR/dCas9 polyplex (b) and MDNP (c) (scale bar: 100 nm). d,e) Fluorescence spectra of MDNP (d) and SDNP (e) after incubated at pH 6.5 and pH 7.4 for 2 h. Cy5–PEI–PBA and Cy3–mPEG_113_‐*b*‐PLys_100_/DMMA or Cy3–mPEG_113_‐*b*‐PLys_100_/SA were employed for the preparation of the MDNP and SDNP, respectively. Excitation wavelength was set at 515 nm. f) Zeta potential variation of MDNP and SDNP with the pH adjustment from pH 8.0 to pH 7.4, and then to pH 6.5. g) CLSM images of the internalization of SDNP and MDNP carrying YOYO‐1‐labeled pDNA (green) at different pHs after 2 h incubation with MDA‐MB‐231 cells. Cell cytoskeleton F‐actin and cell nuclei were counterstained with rhodamine phalloidin (red) and 4,6‐diamino‐2‐phenyl indole (DAPI) (blue), respectively. The scale bars are 20 µm. h,i) Flow cytometry analyses of the cells after incubated with SDNP (h) and MDNP (i) carrying YOYO‐1‐labeled pDNA at pH 7.4 and pH 6.5 for 2 h, respectively. j) Quantification of cell internalization shown by mean fluorescence intensity (MFI). Data in (f) and (j) are presented as mean ± standard deviation (s.d.) from three independent experiments (*n* = 3). The significant levels are shown as ** *p* < 0.01 and *** *p* < 0.001.

MDNP was prepared by first constructing the CRISPR/dCas9 polyplex core via mixing PEI–PBA and CRISPR/dCas9 pDNA at a weight ratio of 6:1 (N/P ratio = 30) in a phosphate buffer saline (PBS), followed by the formation of the shell through the addition of mPEG_113_‐*b*‐PLys_100_/DMMA solution (35 × 10^−9^ m, pH 8.0) to reach a final weight ratio of 6:1 between the polymer and the pDNA (Figure 1a). Instead of PEI with molecular weight of 25 kDa, a PBA‐modified and branched PEI (PEI–PBA) with low molecular weight (*M*_w_ = 2081 Da, average 2.1 PBA per PEI) was employed for the construction of the CRISPR/dCas9 polyplex for a better biocompatibility (Figure S3a,b, Supporting Information). As shown in the gel electrophoresis analysis (Figure S4, Supporting Information) and transfection efficiency tests using plasmid encoding luciferase protein as model pDNA, PEI–PBA achieved the DNA condensation successfully and exhibited acceptable transfection efficiency at N/P ratios ≥ 20 (Figure S5, Supporting Information). Dynamic light scattering (DLS) measurements revealed that the average particle size of the CRISPR/dCas9 polyplex (N/P ratio = 30) was 150.2 ± 6.9 nm, which was then confirmed with transmission electron microscopy (TEM) observation (**Figure** **2**b). Further characterization indicated that the polyplex had a positively charged surface with zeta potential of +22.9 ± 5.8 mV (Figure S6, Supporting Information), suggesting the potentials for cellular internalization and endosomal escape.

Despite the benefits for intracellular transfection of CRISPR/dCas9 plasmid DNA, the PBA‐modified and positively charged surface will activate the immune clearance after administrating into bloodstream, which significantly reduces the overall delivery efficiency to tumor.^18^ To avoid the immune clearance, a layer of anionic polymer, mPEG_113_‐*b*‐PLys_100_/DMMA, was coated onto the CRISPR/dCas9 polyplex core to form MDNP with a PEGylated and negatively charged surface. The polymer was synthesized by ring‐opening polymerization of N6‐carbobenzoxy‐L‐lysine N‐carboxyanhydride (Lys(Z)‐NCA) with PEG—NH_2_ to achieve mPEG_113_‐*b*‐PLys_100_, followed by reacting with DMMA to achieve mPEG_113_‐*b*‐PLys_100_/DMMA (Figures S1 and S2, Supporting Information). The successful coating was confirmed with TEM and DLS measurements, leading to the increase in particle size to 205 ± 10.2 nm (Figure 2c) and the decrease in zeta potential to −12.5 ± 1.6 mV (Figure S6, Supporting Information). Moreover, coating with mPEG_113_‐*b*‐PLys_100_/DMMA reduced the nonspecific protein absorption significantly when incubating the nanoparticles with bovine serum albumin (Figure S7, Supporting Information). As a result, MDNP exhibited enhanced stability in PBS containing 10% fetal bovine serum (FBS) (Figure S8, Supporting Information), suggesting the potential to evade the immune clearance during circulation in blood.^19^

More importantly, acidic environment (pH = 6.5) triggered the breaking of the DMMA groups apart from mPEG_113_‐*b*‐PLys_100_/DMMA, which was first confirmed by monitoring the shiftiness of the characteristic peak attributed to the hydrogen adjacent to amide bond/amino group using ^1^H NMR (Figure 2a). Such conversion effectively changed the anionic polymer mPEG_113_‐*b*‐PLys_100_/DMMA into the cationic polymer mPEG_113_‐*b*‐PLys_100_, leading to the disassembly of the polymer shell from the polyplex core due to the electrostatic repulsion. For better demonstration, a nonresponsive but structurally similar polymer, mPEG_113_‐*b*‐PLys_100_/succinic anhydride (SA) (detailed structure, synthesis method, and characterizations in Figures S1 and S2 in the Supporting Information), was synthesized and coated onto the CRISPR/dCas9 polyplex core to form a comparative nanoparticle (we named it single‐stage delivery nanoparticle, SDNP). The acidic responsiveness of MDNP and SDNP was then investigated via Förster resonance energy transfer (FRET) analysis. This was achieved by first labeling PEI–PBA with Cy5, and mPEG_113_‐*b*‐PLys_100_/DMMA or mPEG_113_‐*b*‐PLys_100_/SA with Cy3, and then recording the fluorescence spectra (excitation wavelength = 515 nm, Cy3) of the MDNP and SDNP made with these labeled polymers, respectively. As shown in the results, obvious FRET signals (Cy5 emission at 662 nm) were observed from both MDNP (Figure 2d, blue) and SDNP (Figure 2e, blue) at pH 7.4, indicating the successful formation of the polymer shell on the polyplex core. By changing pH to 6.5, the FRET signal from MDNP decreased significantly (Figure 2d, red), indicating the disassembly of the polymer shell. In contrast, no obvious change in FRET signal could be observed from SDNP at pH 6.5 (Figure 2e, red), suggesting that the SDNP still maintained the core–shell structure in acidic condition. Further analysis by monitoring the change in zeta potential confirmed that only MDNP presented a significant pH change from −4.8 to +9.5 mV of MDNP over 100 min when changing the pH to 6.5 (Figure 2f), suggesting the disassembly of the shell from MDNP and the exposure of the polyplex core. Considering the different pHs in bloodstream (pH 7.4) and tumor microenvironment (pH 6.5), this responsive shell structure allows MDNP to present different surfaces with the desired physical and chemical properties, respectively, suggesting the great potential of MDNP to overcome physiological barriers to achieve efficient delivery of CRISPR/dCas9 system to tumor cells.

### 2.2 Cellular Internalization of MDNP and Endosomal Escape of pDNA

For an effective CRISPR/dCas9‐based cancer treatment, it is crucial for MDNP to internalize into cancer cells efficiently. By preparing MDNP with YOYO‐1‐labeled pDNA and then incubating with cancer cells (MDA‐MB‐231), the cellular uptake behaviors of MDNP were observed directly with confocal laser scanning microscope (CLSM). SDNP was also employed in this study for the comparison. According to the CLSM images (Figure 2g), an obvious uptake of DNA was observed from the cells incubated with MDNP at pH 6.5. In contrast, much lower level of uptake could be observed from the cells treated with MDNP at pH 7.4, as well as those incubated with SDNP at both pHs 6.5 and 7.4. These observations were confirmed with flow cytometry analysis (Figure 2h for MDNP, and Figure 2i for SDNP). Quantitative analysis of the flow cytometry results (Figure 2j) indicated that negligible differences in uptake efficiency could be observed from the cells incubated with SDNP at different pHs, whereas a significant enhanced uptake efficiency was observed from the MDNP‐treated cells when incubating at pH 6.5. This result confirmed the pH‐responsive capability of MDNP, indicating that the acidic environment triggered the dissociation of the polymer shell of MDNP and the exposure of the cationic polyplex. Additional comparison with PEI_25k_/pDNA (pH 6.5) indicated that MDNP exhibited 1.9‐fold higher cellular uptake efficiency, which could be caused by the PBA groups conjugated on the surface of the polyplex.

Effective endosomal escape is another necessary step for a successful delivery of CRISPR/dCas9 system. Since the polyplex core of MDNP was made from PEI–PBA, it was expected to effectively escape from the endo‐/lysosomes and release pDNA into cytoplasm due to the protonation of PEI–PBA that disrupted the endo‐/lysosomes.^20^ To investigate the endosomal escape capability, MDNP carrying TOTO‐3‐labeled pDNA was exposed to MDA‐MB‐231 cells (cultured at pH 6.5), and the cells were then observed using CLSM after 1, 2, and 4 h incubation. Before the observation, the late endosomes and lysosomes of the cells were stained with LysoTracker Green. As shown in the fluorescence images (Figure S9a, Supporting Information), the colocalizations (yellow) of TOTO‐3‐labeled pDNA and late endo‐/lysosomes were observed after 2 h incubation, indicating the entrapment of the pDNA in late endosomes and lysosomes. Following another 2 h incubation, the fluorescence signal of the pDNA (red) was clearly observed, implying the successful endosomal escape of the pDNA delivered by MDNP. Further calculation of the overlap coefficient (*R*) (Figure S9b, Supporting Information) confirmed the CLSM observation, indicating the decrease in the colocalizations of the endo‐/lysosomes and the pDNA after 4 h incubation. These results confirmed the capability of MDNP to escape from endo‐/lysosomes and deliver the pDNA into the cytoplasm, allowing the effective expression of pDNA in the targeted cells.

### 2.3 Transfection Efficiency of MDNP in Cancer Cells

**Figure 3:**
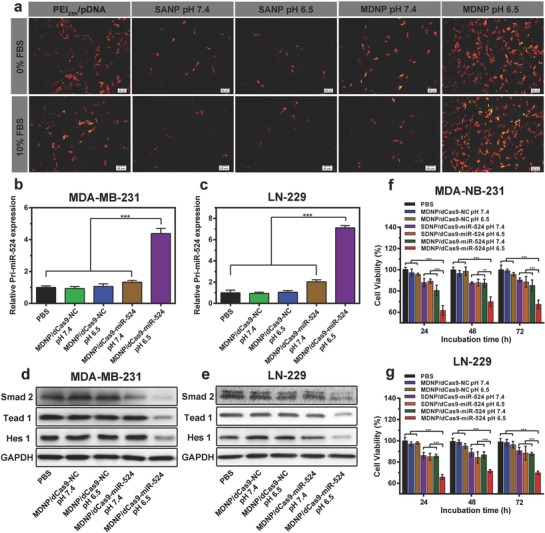
a) Fluorescence microscope images of LN‐229 cells transfected with MDNP carrying pDNA encoding tdTomato fluorescent protein (orange) in culture media with 0% or 10% serum, respectively. The scale bar is 50 µm. b,c) Relative expression levels of Pri‐miR‐524 in MDA‐MB‐231 (b) and LN‐229 (c) cells after treating with MDNP. The expression of Pri‐miR‐524 was detected by quantitative real‐time PCR assay. d,e) Western blot analysis of the Smad2, Tead1, and Hes1 expressions in MDA‐MB‐231 (d) and LN‐229 (e) cells after treating with MDNP. Glyceraldehyde 3‐phosphate dehydrogenase (GAPDH) was used as a loading control. f,g) Cell viability of MDA‐MB‐231 (f) and LN‐229 (g) cells after treating with MDNP at pH 7.4 and pH 6.5 for 24, 48, and 72 h incubation. Cell viability was assessed using CCK‐8 assay. All data in (b), (c), (f), and (g) are presented as mean ± s.d. from three independent experiments (*n* = 3). The significant levels are shown as ** *p* < 0.01 and *** *p* < 0.001.

Since efficient dCas9 protein expression is a prerequisite for CRISPR/dCas9‐based cancer treatment, we studied the gene transfection efficiency of MDNP in LN‐229 cell line. For the easier observation, pDNA that expresses tdTomato fluorescent protein was employed for studying the transfection efficiency. As shown in **Figure** **3**a, very low level of tdTomato expressions was observed from the SDNP‐treated LN‐229 cells (at pH 6.5 and pH 7.4) and the MDNP‐treated cells at pH 7.4, which was caused by the inefficient cellular uptake of the nanoparticles. In contrast, high level expression of tdTomato was observed in the cells treated with MDNP at pH 6.5, and the portion of tdTomato‐positive cells was higher than that of the cells treated with PEI_25k_/pDNA, which agreed with the results from the cell uptake analysis (Figure 2j). More importantly, the introduction of 10% FBS did not impact the transfection efficiency of MDNP significantly, whereas the efficiency of PEI_25k_/pDNA was reduced in the same condition (Figure 3a and Figure S10 (Supporting Information)). This is because the polyplex core of MDNP facilitated the transfection of pDNA using low molecular weight PEI–PBA, resulting in less nonspecific protein absorption and stronger binding to cell membrane via PBA–sialic acid complexation compared with PEI_25k_. Further transfection studies using luciferase‐expressing pDNA yielded a similar result (Figure S11, Supporting Information). With all these evidences, we can conclude that MDNP could efficiently transfect pDNA into cancer cells to express the genes of interest. Successful transfection in the presence of serum further suggests the potential of MDNP to achieve efficient gene transfection in vivo, which is essential for CRISPR/dCas9‐based cancer gene therapy.

### 2.4 CRISPR Activation of miR‐524 Expression with MDNP in Cancer Cells

Recent research has identified that miR‐524 is usually suppressed in many types of cancer cells. Overexpression of this miRNA can restrain the proliferation and migration of cancer cells, which could potentially benefit to the overall survival of cancer patients.^21^ Upregulation of miR‐524 expression using MDNP was performed on two cancer cell lines (MDA‐MB‐231 and LN‐229) to investigate the ability of MNDP to induce CRISPR/dCas9‐based gene transcriptional regulation and eventually suppress the proliferation of cancer cells. To this end, a pDNA (we named it dCas9–miR‐524), which expresses dCas9 activator (dCas9VP64) and a sgRNA that targets the promoters or enhancers of the primary transcription content of miR‐524 (Pri‐miR‐524), was first constructed (detailed method in Figure S12 in the Supporting Information). A nonfunctional expression vector (named NC) was also employed as the negative control for the following studies. MDNP carrying dCas9–miR‐524 (MDNP/dCas9–miR‐524) was incubated with the cells (MDA‐MB‐231 and LN‐229, respectively) for 2 h. The cells were then rinsed with PBS for 3 times and for further cultured with fresh medium for 48 h. The expression level of Pri‐miR‐524 was analyzed via quantitative real‐time polymerase chain reaction (qRT‐PCR). For comparison, MDNP carrying NC (MDNP/NC) was also employed to perform the same study. According to the results (Figure 3b,c), the expression levels of Pri‐miR‐524 were significantly upregulated to 438% in MDA‐MB‐231 and 711% in LN‐229 when incubating with MDNP/dCas9–miR‐524 at pH 6.5 (100% for untreated cells). In contrast, no significant upregulation of Pri‐miR‐524 expression could be observed from the cells treated with SDNP carrying dCas9–miR‐524 (SDNP/dCas9–miR‐524), no matter at pH 7.4 or pH 6.5 (Figure S13, Supporting Information). This result indicated that MDNP could only achieve the CRISPR activation in acidic environment, which effectively minimizes the Pri‐miR‐524 upregulation in tissues other than tumor and thus reduce the potential side effects.

The expression of miR‐524 can restrain the proliferation of cancer cell via targeting and inhibiting the expression of Smad2, Hes1, and Tead1 which are essential proteins for transforming growth factor‐β (TGF‐β), Norch, and Hippo signaling pathways,^21^, ^22^ respectively. For this reason, upregulation of endogenous miR‐524 expression should decrease the expression level of these target proteins, and eventually inhibit the proliferation of the cancer cells. The expression of Smad2, Hes1, and Tead1 was accessed via Western blot analysis after treating the cancer cells with MDNP in the abovementioned conditions. As shown in Figure 3d,e, the expression levels of these three proteins were reduced significantly in the cells treated with MDNP at pH 6.5, whereas no differences in the protein expression levels could be observed from other groups compared to the untreated cells. This result is in agreement with the expression level of Pri‐miR‐524, which confirmed again the successful CRISPR activation mediated by MDNP. Moreover, this result also confirmed the capability of MDNP/dCas9–miR‐524 to regulate multiple oncogenic pathways synergistically via the transcriptional control of endogenous miR‐524, suggesting the great potential of MDNP/dCas9–miR‐524 in repressing tumor growth.

To evaluate the antitumor effect of MDNP/dCas9–miR‐524, cancer cells (MDA‐NB‐231 and LN‐229) were incubated with MDNP/dCas9–miR‐524, SDNP/dCas9–miR‐524, and MDNP/NC at pHs 6.5 and 7.4, respectively. The cell viabilities were then measured using cell counting kit‐8 (CCK‐8) after 24, 48, and 72 h incubation. Figure 3f,g summarizes the relative cell viabilities after the incubation (100% for untreated cells at 24, 48, and 72 h, respectively). Obviously, the cells incubated with MDNP/dCas9–miR‐524 at pH 6.5 exhibited significant lower viability (64% at most) than any other comparative groups, suggesting the antiproliferative capacity of MDNP/dCas9–miR‐524 that can be only activated in acidic environment. Moreover, negligible loss of viability was observed from the cells treated with MDNP/NC (Figure 3f,g), indicating that MDNP itself was nontoxic and thus the loss of viability of MDNP/dCas9–miR‐524‐treated cells was caused by the CRISPR activation of miR‐524 expression.

### 2.5 Tumor‐Targeting Capability of MDNP in Mice

**Figure 4:**
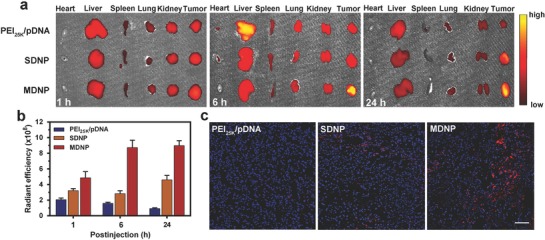
a) Ex vivo fluorescence images of isolated tissues from the MDA‐MB‐231 tumor‐bearing mice after intravenous injection of PEI_25k_/pDNA, SDNP, and MDNP carrying TOTO‐3‐labeled pDNA (red) at 1, 6, and 24 h postinjection. b) Quantitative analysis of the tumor accumulation of the pDNA based on the fluorescence intensity from the ex vivo images. c) CLSM images of the tumor sections from the mice 24 h postinjection. The cell nuclei were stained with DAPI (blue), and the pDNA was stained with TOTO‐3 (red). The scale bar is 100 µm. Data in (b) are presented as mean ± s.d. from three independent experiments (*n* = 3).

For an effective CRISPR/dCas9‐based cancer gene therapy, it is essential to deliver the CRISPR/dCas9 system to tumor tissue after the administration. MDNP is specially designed for the in vivo delivery of CRISPR/dCas9 system to overcome delivery barriers and accumulate in tumor tissue. To investigate on the tumor accumulating ability of MDNP, pDNA was stained with TOTO‐3 and loaded into MDNP. The MDNP was then injected into tumor‐bearing (MDA‐MB‐231) mice through tail vein. All experimental protocols were conducted within Tianjin Medical University guidelines for animal research and were approved by Institutional Animal Care and Use Committee. At different time points, the mice were sacrificed, and the major organs and the tumors were collected for ex vivo evaluation. **Figure** **4**a compares the accumulation of the pDNA delivered via different systems. As shown in the ex vivo images, pDNA delivered with PEI_25k_ failed to reach the tumor, resulting in a rapid accumulation in liver 6 h postinjection and the subsequent clearance from major organs within 24 h. In contrast, both SDNP and MDNP achieved the delivery of pDNA into tumor after the intravenous injection. Considering the nanoscaled size of SDNP and MDNP, the tumor accumulation should be caused by the enhanced permeability and retention (EPR) effects.^23^ Compared to SDNP, MDNP exhibited significantly higher level of pDNA in tumor at 6 h postinjection, suggesting a much faster accumulation. This was caused by the dissociation of the PEGylated shell and the exposure of the polyplex core of MDNP in response to the acidic tumor microenvironment. Since the polyplex core of MDNP possesses acationic surface with PBA groups, the affinity between the nanoparticle and the tumor tissue was enhanced significantly,^16^ leading to a more efficient accumulation. Quantitative analysis of the tumor accumulation from the ex vivo images (Figure 4b) indicated that delivering via MDNP achieved a significantly higher level of pDNA in tumor compared to those delivered with SDNP and PEI_25k_ especially at 6 and 24 h postinjection. Moreover, negligible differences in the radiant efficiency of the pDNA in tumor could be observed between 6 and 24 h when delivered using MDNP, suggesting that the exposure of the polyplex core of MDNP might further enhance the penetration and intracellular uptake of the pDNA. CLSM observation of the tumor sections (Figure 4c) indicated a remarkably higher level of pDNA throughout the tumor tissue from the mice treated with MDNP, suggesting the high delivery efficiency of MDNP in tumor‐targeted delivery of pDNA.

### 2.6 Tumor Growth Inhibition with MDNP/dCas9–miR‐524

**Figure 5:**
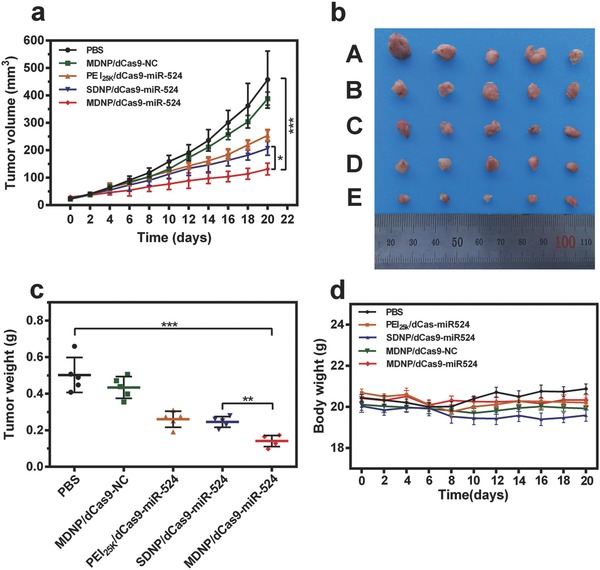
a) Tumor growth curves of the mice injected with PBS, MDNP/NC, PEI_25k_/dCas9–miR‐524, SDNP/dCas9–miR‐524, MDNP/dCas9–miR‐524, respectively. b) Ex vivo observation of the tumors from the treated mice 20 days postinjection (A: PBS, B: MDNP/NC, C: PEI_25k_/dCas9–miR‐524, D: SDNP/dCas9–miR‐524, E: MDNP/dCas9–miR‐524). c) Comparison of the weight of the tumors from the mice after treatment. d) Changes in body weight after treating the mice with different formulations. All data in (a), (b), (c), and (d) are presented as mean ± s.d. from five independent experiments (*n* = 5). The significant levels are shown as * *p* < 0.05, ** *p* < 0.01, and *** *p* < 0.001.

**Figure 6:**
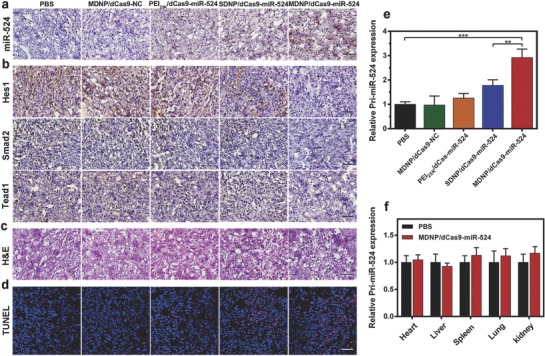
a) RNA in situ hybridization presenting the expression level of miR‐524 in the tumor tissue from the mice in each treatment group. Cell nuclei are stained blue, and miR‐524 are stained brown. The scale bar is 100 µm. b) Immunohistochemistry analyses of the expression of Hes1, Tead1, and Smad2 in each treatment group, nuclei are stained blue, and the proteins are stained brown. The scale bar is 100 µm. c,d) H&E staining (c) and TUNEL (d) analysis of the tumor tissues from the mice in each treatment group. The scale bar is 100 µm. In TUNEL staining, normal cell nuclei are stained blue and apoptotic cell nuclei are stained red. The scale bar is 100 µm. e,f) Relative expression levels of Pri‐miR‐524 in tumors (e) and nontargeted organs (f) from the mice treated with MDNP/dCas9–miR‐524 and PBS, respectively. All data in (e) and (f) are presented as mean ± s.d. from five independent experiments (*n* = 5). The significant levels are shown as ** *p* < 0.01 and *** *p* < 0.001.

Based on the successful tumor‐targeted delivery of pDNA, additional studies were performed to investigate the potential of MDNP in CRISPR/dCas9‐based cancer gene therapy. MDNP/dCas9–miR‐524 was injected into tumor‐bearing mice (MDA‐MB‐231) via tail vein every 3 days for 20 days. For better comparison, comparative groups, including PBS, MDNP/NC, PEI_25k_/dCas9–miR‐524, SDNP/dCas9–miR‐524, were employed to perform the same study. A continuous monitoring of the tumor volumes during the 20 days (**Figure** **5**a) indicated that the tumors from the mice receiving MDNP/dCas9–miR‐524 grew significantly slower than those with other treatments. This result was further confirmed by the ex vivo observation of the tumors (Figure 5b) and the comparison of the tumor weights (Figure 5c). Moreover, negligible variations of body weights were observed from the mice treated with MDNP (Figure 5d), suggesting the good biocompatibility of MDNP. Additionally, no elevation in inflammatory cytokine and immune globulin (Ig) secretion was observed from the mice treated with MDNP after the administration (Figures S14 and S15, Supporting Information), suggesting the low immunogenicity of MDNP. Further studies on the tumor tissues with RNA in situ hybridization (RISH) and immunohistochemistry (IHC) analyses confirmed that the tumor growth inhibition was associated with the CRISPR/dCas9–miR‐524‐mediated gene transcription regulation. According to the RISH and IHC analyses, the tumor from the MDNP/dCas9–miR‐524‐treated mice exhibited remarkably higher miR‐524 expression level (**Figure** **6**a), which led to significant inhibition of the expression of Smad2, Hes1, and Tead1 (Figure 6b). By inhibiting the essential proteins for TGF‐β, Norch, and Hippo signaling pathways, MDNP/dCas9–miR‐524 effectively triggered the apoptosis of the tumor cells,^21^, ^22^ which was confirmed by the direct observation of the slices stained with hematoxylin and eosin (H&E) and terminal deoxynucleotidyl transferase 2′‐deoxyuridine 5′‐triphosphate (dUTP) nick end labeling (TUNEL) (Figure 6c,d), respectively. The effective inhibition of tumor growth confirmed the feasibility of applying CRISPR/dCas9–miR‐524 system in vivo for cancer treatment with the aid of aproperly designed delivery system. Furthermore, the relative expressions of Pri‐miR‐524 levels in tumors and normal organs were analyzed by qRT‐PCR. As shown in Figure 6e, the tumors from the mice treated with MDNP/dCas9–miR‐524 exhibited significantly elevated Pri‐miR‐524 levels (2.92‐fold higher than those from the mice treated with PBS), suggesting the successful upregulation by MDNP/dCas9–miR‐524. More importantly, upregulation of Pri‐miR‐524 was not observed from the nontargeted organs (e.g., heart, liver, spleen, lung, and kidney) of the mice treated with MDNP/dCas9–miR‐524 (Figure 6f), suggesting the reduced off‐target effects that may cause potential side effects. Considering the presence of the target gene of dCas9–miR‐524 in both the tumors and these nontargeting organs, such tumor targeting performance has to be attributed to the tumor‐targeting capability of MDNP as well as the responsive release of dCas9–miR‐524 from MDNP stimulated by the acidic tumor microenvironment. With the remarkable antitumor effect, reduced off‐target effect, and low immunogenicity, MDNP/dCas9–miR‐524 presented its potential in the development of novel CRISPR/dCas9‐based cancer gene therapies.

## 3 Discussion

CRISPR/dCas9 system is one of the most powerful tools for the precise and efficient control of endogenous gene expression.^2^ Unlike CRISPR/Cas9 systems that require cutting and mutating the genome, CRISPR/dCas9 regulates the endogenous gene expression at transcription level.^2^ Since tumor growth can be relieved by suppressing oncogenes or upregulating tumor suppressor genes,^6^ CRISPR/dCas9 holds great potential for the development of safe and effective anticancer therapeutics.^4^ However, lack of efficient delivery systems, especially for the in vivo tumor‐targeted delivery systems, prevents its clinical translation. For a successful CRISPR/dCas9‐based cancer treatment in vivo, it is required for a delivery system to meet several design criteria including 1) a negatively charged and antiprotein‐fouling surface to evade the blood clearance during circulation, and 2) a positively charged and membrane‐binding surface to facilitate the intracellular transport of CRISPR/dCas9 system to allow it to function normally.^12^ However, those requirements seem conflicting with each other, preventing the construction of an ideal delivery system for CRISPR/dCas9 systems.

After a careful analysis of the delivery route, we realized that a tumor‐targeted delivery of CRISPR/dCas9 had to experience three delivery stages, including circulating in blood after the administration (Stage 1), reaching and accumulating in tumor tissues (Stage 2), internalizing into tumor cells (Stage 3). The markedly different microenvironments between blood circulation and tumor tissues offer us the opportunity to simultaneously achieve all the desired features with one delivery system to overcome all the major delivery barriers of CRISPR/dCas9 systems. In this report, we present a MDNP which displays different surface properties in response to the change of the surrounding microenvironment. To achieve this feature, the MDNP is designed as a core–shell structure, in which the core is a PBA‐modified cationic polyplex and the shell is made of a PEG‐based responsive polymer (Figure 1a). By administration and circulation in blood (Stage 1), MDNP maintains a stable core–shell structure with a negatively charged and PEGylated surface, which is arguably the most practical method to enhance the circulation stability of nanoparticles. Due to the EPR effects, MDNP enters tumor tissues eventually (Stage 2). The acidic tumor microenvironment triggers the dissociation of the polymer shell from the MDNP, leading to the exposure of the cationic polyplex. Since the cationic surface has a much stronger electrostatic attraction to tumor tissues, the polyplex will stay and accumulate in tumor tissues. Moreover, the polyplex was modified with PBA groups, which bind to the sialic acids that are usually overexpressed by cancer cells and enhance the intracellular uptake of the polyplex (Stage 3). With this design, MDNP can present different surface properties in the different delivery stages, which fulfills all the requirements simultaneously for achieving an efficient delivery of CRISPR/dCas9 system.

The delivery efficiency of MDNP was evaluated both in vitro and in vivo using fluorescence‐labeled pDNA as a model. A significant enhancement in cellular uptake of the pDNA was observed when delivering with MDNP in acidic condition (pH 6.5) compared to the uptake in neutral condition (pH 7.4) (Figure 2g,j). This result confirms the acid‐triggered dissociation of the MDNP shell, indicating that MDNP is capable to evade the cellular uptake under neutral pH (e.g., in blood), while internalizes into cells effectively under acidic pH (e.g., in tumor tissues). In mice, biodistribution analysis confirmed this result, indicating a significantly higher tumor‐targeting efficiency of MDNP than other delivery carriers (Figure 4a,b). Less liver accumulation was also observed when delivering pDNA with MDNP compared to that delivered with PEI_25k_, suggesting the effective immune clearance evasion due to PEGylated surface. Moreover, MDNP also exhibited faster and more efficient tumor targeting compared to SDNP, indicating that the dissociation of the PEGylated shell of MDNP and the exposure of the cationic polyplex in response to the acidic tumor microenvironment did enhance the tumor accumulation, which eventually increase the uptake of the pDNA by cancer cells (Figure 4c).

As a delivery system specially designed for CRISPR/dCas9‐based cancer gene therapy, the performance of CRISPR/dCas9‐based gene expressing regulation and antitumor effects were investigated both in cell and in mice. In these studies, we employed dCas9–miR‐524 as the CRIPSR/dCas9 system since the successful delivery of dCas9–miR‐524 could activate the expression of miR‐524, which then inhibited the expressions of three essential proteins related to cancer cell proliferation. Consistent with the delivery efficiency studies, increased expression level of miR‐524 was observed in the cells treated with MDNP/dCas9–miR‐524 in acidic condition (Figure 3b,c), and the eventual downregulation of Smad2, Hes1, and Tead1 (Figure 3d,e) led to the significant loss in cell viability when incubating cancer cells with MDNP (Figure 3f,g). Administration of MDNP/dCas9–miR‐524 to tumor‐bearing mice resulted in a remarkable inhibition in tumor growth (Figure 5a–c), which was caused by the activation of miR‐524 expression that led to the apoptosis of tumor cells (Figure 6c,d). These results confirmed the successful in vivo CRISPR/dCas9‐based gene expressing regulation achieved using MDNP, suggesting the potential of MDNP/dCas9–miR‐524 as an effective strategy for the development of CRIPSR/dCas9‐based cancer gene therapy.

## 4 Conclusion

In conclusion, we have demonstrated a multistage delivery nanoparticle that can deliver CRISPR/dCas9 systems and facilitate effective regulation on gene expression both in vitro and in vivo. Systemic administration of MDNP/dCas9–miR‐524 to tumor‐bearing mice presented remarkable effects on tumor growth inhibition, suggesting the feasibility to utilize MDNP to achieve tumor‐targeting delivery of CRISPR/dCas9 with sufficient level to realize its therapeutic effects. More importantly, the microenvironment‐responsive polymer shell endows MDNP with the capability to present different surface properties at different delivery stages, allowing the MNDP to overcome multiple physiological barriers and deliver the payload to tumor tissues with an optimal efficiency. With these capabilities, MDNP could become a fundamental technology to address the delivery problems in the development of CRISPR/dCas9‐based cancer gene therapy. More broadly, MNDP could be also adapted to deliver other types of CRISPR systems to raise exciting opportunities for novel CRISPR‐based cancer treatments.

## 5 Experimental Section

*Synthesis of PEI–PBA*: The synthesis of PEI–PBA was achieved by conjugating 2‐bromomethylphenylboronic acid onto branched PEI. Briefly, 1.80 g of PEI (*M*_w_ = 1.8 kDa) was first dissolved in methanol to reach a concentration of 120 mg mL^−1^, and then 0.54 g of 2‐bromomethylphenylboronic acid was added. The reaction solution was stirred under reflux at 70 °C for 12 h, and then the product was precipitated by dropping the reaction solution into cold ether. The successful conjugation was confirmed using ^1^H NMR analysis, indicating that the molecular weight of the PEI–PBA was 2068 Da. Detailed synthesis procedures and characterizations are provided in the Supporting Information.

*Synthesis of mPEG_113_‐b‐PLys_100_/DMMA and mPEG_113_‐b‐PLys_100_/SA*: The synthesis of mPEG_113_‐*b*‐PLys_100_/DMMA was achieved by conjugating DMMA onto mPEG_113_‐*b*‐PLys_100_ (detailed synthesis procedures and characterizations are provided in the Supporting Information) Briefly, 100 mg of mPEG_113_‐*b*‐PLys_100_ was dissolved in sodium bicarbonate buffer (pH 8.5, 50 × 10^−3^ m) to reach a concentration of 10 mg mL^−1^, and then 211.2 mg of DMMA (five equivalents to the amino groups of mPEG_113_‐*b*‐PLys_100_) was added. After the action, unreacted DMMA was removed by dialysis, and mPEG_113_‐*b*‐PLys_100_/DMMA was obtained by lyophilization. The synthesis of mPEG_113_‐*b*‐PLys_100_/SA was similar to that of mPEG_113_‐*b*‐PLys_100_/DMMA by replacing DMMA with SA. The successful synthesis was confirmed using ^1^H NMR analysis that ≈90% of the amine groups on mPEG_113_‐*b*‐PLys_100_ reacted with DMMA or SA. Detailed synthesis procedures and characterizations are provided in the Supporting Information.

*Preparation of MDNP and SDNP*: The MDNP and SDNP were prepared by mixing mPEG_113_‐*b*‐PLys_100_/DMMA and mPEG_113_‐*b*‐PLys_100_/SA solutions with the solution of PEI–PBA/pDNA polyplex, respectively. First, the PEI–PBA (0.1 mL, 1.5 mg mL^−1^ in water) and dCas9–miR‐524 pDNA (0.1 mL, 250 µg mL^−1^ in water) were mixed gently and incubated for 15 min to form the PEI–PBA/pDNA polyplex. Then, the mPEG_113_‐*b*‐PLys_100_/DMMA (0.1 mL, 3 mg mL^−1^) and mPEG_113_‐*b*‐PLys_100_/SA (0.1 mL, 3 mg mL^−1^) were added to the solution of PEI–PBA/pDNA polyplex (0.1 mL) and incubated for another 15 min to form MDNP and SDNP, respectively.

*In Vitro CRISPR Activation of miR‐524 Expression with MDNP in Cancer Cells*: qRT‐PCR and Western blot were performed to study in vitro CRISPR activation of miR‐524 expression with MDNP in cancer cells. Briefly, MDA‐MB‐231 and LN‐229 cells were seeded into 6‐well plates at a density of 2 × 10^5^ cells per well and incubated overnight in 2 mL Dulbecco's modified Eagle medium (DMEM) with 10% FBS v/v. Before the transfection, the culture medium was replaced with the fresh ones and adjusted to pHs 7.4 and 6.8, respectively, following by the addition of 100 µL of MDNP solution (3 µg dCas9–miR‐524 pDNA per well). After 4 h incubation, the culture medium was removed, and the cells were cultured with 2 mL fresh medium for further 48 h. The RNA and the proteins were extracted for qRT‐PCR and Western blot analysis. More detailed procedures are provided in the Supporting Information.

*In Vitro Cytotoxicity Analysis*: The in vitro antitumor effect was studied by evaluate the viability of the cancer cells (MDA‐MB‐231 and LN‐229) after treating with MDNP/dCas9–miR‐524 at pH 7.4 and pH 6.5. Briefly, cells were seeded into 96‐well plates at a density of 5 × 10^3^ cells per well and incubated overnight in 100 µL DMEM with 10% FBS v/v. Before transfection, the culture medium was replaced with 100 µL fresh ones and adjusted to pHs 7.4 and 6.8, respectively. 10 µL of the MDNP was added into the cell cultures to reach 200 ng pDNA per well. After 4 h incubation, the culture medium was removed, and the cells were cultured in fresh medium for another 24, 48, and 72 h, respectively. For the comparison, PEI_25k_/pDNA polyplex and MDNP/NC were employed to perform the same studies. After the treatment, cell viability was assessed using CCK‐8 assay. The cell viability was calculated by referring to the control group without any treatment.

*In Vivo Distribution and Imaging*: The tumor‐bearing mice were generated by subcutaneous injection of MDA‐MB‐231 cells (5 × 10^6^ for each mouse) in the mammary fat pad, and the mice were randomly divided into three groups. When the tumor volume was about 400 mm^3^, three groups of the mice were intravenously injected with 100 µL of PEI_25k_/pDNA polyplex, SDNP, and MDNP, all of which contained 10 µg TOTO‐3‐labeled pDNA, respectively. The distribution of the pDNA was imaged using IVIS Lumina imaging system (Caliper Life Sciences, USA) at 1, 6, and 24 h postinjection. The results were analyzed using Living Image 3.1 software (Caliper Life Sciences). To determine the distribution of TOTO‐3‐labeled pDNA in tumor, the tumor tissues were fixed, and the tissue sections were observed using a CLSM (Olympus, FV1000). More detailed procedures are provided in the Supporting Information.

*In Vivo Tumor Inhibition*: The tumor‐bearing mice were established as described above. When the tumor volume was around 25 mm^3^ at 10 days after the cell implantation, the mice were randomly divided into five groups (five mice per group) and intravenously injected with 100 µL of PBS, MDNP/dCas9–NC, PEI_25k_/dCas9–miR‐524, SDNP/dCas9–miR‐524, and MDNP/dCas9–miR‐524 containing 10 µg pDNA per mouse every 3 days, respectively. Tumor growth was monitored by measuring the perpendicular diameter of the tumor using calipers. The estimated volume was calculated according to the formula: tumor volume (mm^3^) = 0.5 × length × width^2^. After finishing the treatment, the tumors were harvested from the mice. The expression of miR‐524 in tumor tissues was detected by qRT‐PCR and RISH. IHC was performed for analyzing the expression levels of Hes1, Tead1, and Smad2. For the observation of tumor cell apoptosis, tumor slices were stained with H&E and TUNEL, respectively. All the images were recorded using CLSM (Olympus, FV1000) or a fluorescence microscope (Olympus, CX41). More detailed procedures are provided in the Supporting Information.

## Conflict of Interest

The authors declare no conflict of interest.

## Supporting information

Supplementary
