# Supplementary material for: Multistage Delivery Nanoparticle Facilitates Efficient CRISPR/dCas9 Activation and Tumor Growth Suppression In Vivo
Source: Adv Sci (Weinh). 2018 Oct 25;6(1):1801423. doi: 10.1002/advs.201801423 (PMC6325604; doi:10.1002/advs.201801423)
Supplement: Supplementary file 1 — Supplementary [file ADVS-6-1801423-s001.pdf]

Copyright WILEY-VCH Verlag GmbH & Co. KGaA, 69469 Weinheim, Germany, 2018.

ADVANCED SCIENCE Open Access

Supporting Information

for *Adv. Sci.,* DOI: 10.1002/advs.201801423

Multistage Delivery Nanoparticle Facilitates Efficient CRISPR/dCas9 Activation and Tumor Growth Suppression In Vivo

Qi Liu, Kai Zhao, Chun Wang, Zhanzhan Zhang, Chunxiong Zheng, Yu Zhao, Yadan Zheng, Chaoyong Liu, Yingli An, Linqi Shi, Chunsheng Kang,* and Yang Liu**

Supporting Information

Multistage Delivery Nanoparticle Facilitates Efficient CRISPR/dCas9 Activation and Tumor Growth Suppression In Vivo

Qi Liu, Kai Zhao, Chun Wang, Zhanzhan Zhang, Chunxiong Zheng, Yu Zhao, Yadan Zheng, Chaoyong Liu, Yingli An, Linqi Shi, Chunsheng Kang*, Yang Liu**

Q. Liu, C. Wang, Z. Zhang, C. Zheng, Y. Zhao, Y. Zheng, Y, An, Prof. L. Shi, Prof. Y. Liu

State Key Laboratory of Medicinal Chemical Biology

Key Laboratory of Functional Polymer Materials of Ministry of Education

College of Chemistry

Nankai University

Tianjin, 300071, China

E-mail: shilinqi@nankai.edu.cn; yliu@nankai.edu.cn

K. Zhao, C. Liu, Prof. C. Kang

Tianjin Neurological Institute

Department of Neurosurgery

Tianjin Medical University General Hospital

Tianjin, 300052, China.

E-mail: kang97061@tmu.edu.cn

Table of Content

Materials 4

Synthesis of PEI-PBA..4

Synthesis of $mPEG_{113}$-*b*-$PLys_{100}$ 5

Synthesis of $mPEG_{113}$-*b*-$PLys_{100}$/DMMA and $mPEG_{113}$-*b*-$PLys_{100}$/SA...................................6

Investigation on the pH-responsiveness of $mPEG_{113}$-*b*-$PLys_{100}$/DMMA.................................6

Preparation of MDNP and SDNP 6

Electrophoresis, DLS, and TEM analysis of MDNP and SDNP 7

Evaluation of the non-specific protein adsorption and stability of MDNP 8

Fluorescence resonance energy transfer (FRET) assay 8

Cell culture..8

Analyses of cellular uptake and endosomal escape 9

In vitro gene transfection 10

Quantitative real-time PCR assay evaluating the activation of miR-524 expression 10

Western blotting analysis...11

In vitro cytotoxicity analysis..11

In vivo distribution of MDNP 12

Immunogenicity evaluations assessed with inflammatory cytokine secretion 13

In vivo tumor growth inhibition by MDNP...13

Detection of Pri-miR-524 expression in tumors and normal organs 14

RNA *in situ* hybridization detection of miR-524 in tumor tissues 14

Immunohistochemistry analysis and H&E staining..14

Statistical Analysis...15

Table of Figures

Figure S1. Synthesis routes of the polymers 16

Figure S2. ^{1}H NMR spectra of the polymers 17

Figure S3. Cytotoxicity of the polymers 18

Figure S4. The capability of PEI-PBA to condense pDNA 19

Figure S5. Luciferase expression of PEI-PBA/pDNA transfected in LN-229 cells at different N/P ratios 20

Figure S6. Zeta potentials of PEI-PBA/pDNA polyplex, SDNP and MDNP 21

Figure S7. BSA adsorption of PEI-PBA/pDNA polyplex and MDNP 22

Figure S8. Stability of SDNP and MDNP in PBS containing 10% FBS 22

Figure S9. Endosomal escape of MDNP 23

Figure S10. *In vitro* transfection efficiency of SDNP and MDNP at different pH in LN-229 cells 24

Figure S11. *In vitro* transfection efficiency of the SDNP and MDNP in LN-229 cells 25

Figure S12. Schematic diagram of dCas9-miR-524 system. 26

Figure S13. Relative expression levels of Pri-miR-524 in cells transfected with SDNP/dCas9-miR-524 27

Figure S14. Plasma cytokine levels after injection of MDNP 28

Figure S15. The levels of IgE, IgM, IgG after injection of MDNP. 29

Materials. Reagents and solvents were purchased from Sigma-Aldrich (Shanghai, china) and used as received without further purification unless otherwise noted. Dialysis membranes were purchased from Tian Nan Science and Technology (Tianjin, China). All the cell lines, including human glioma (LN-229) and human breast cancer (MDA-MB-231), were purchased from American Type Culture Collection (ATCC). The Dulbecco's Modified Eagle Medium (DMEM) growth medium, fetal bovine serum (FBS) and penicillin/streptomycin were purchased from Gibco (Thermo Fisher, USA). Linear polyethylenimine 25 kDa (PEI25K) and branched polyethylenimine 1.8 kDa (PEI1.8K) were purchased from Alfa Aesar (Shanghai, China). YOYO-1, TOTO-3, rhodamine phalloidin, LysoTracker® Green, paraformaldehyde, 4,6-diamidino-2-phenylindole dihydrochloride (DAPI), and Lipofectamine 2000 were purchased from Invitrogen (USA). Cy5-NHS and Cy3-NHS were obtained from Oukainasi Technology (Beijing, China). Bicinchoninic acid (BCA) protein assay kit was obtained from Solarbio Science & Technology (Beijing, China). Cell Counting Kit-8 (CCK-8) was obtained from Zhuangmeng Biotech (Beijing, China). Fluorescent TUNEL staining kit was obtained from Zhongshanjinqiao Biotech (Beijing, China). ABC-peroxidase, diaminobenzidine (DAB) and miRNA In Situ Hybridization (RISH) kit were purchased from Bersin Biotech (Guangzhou, China). Hairpin-it miRNA qPCR Quantitation Kit was obtained from GenePharma Biotech (Shanghai, China). All ELISA kits was purchase from Jianglai biotech (Shanghai, China). All the antibodies were purchased from Santa Cruz Biotech (Shanghai, China). EndoFree Plasmid Kit was purchased from Qiagen (USA). Luciferase reporter gene assay kit were purchased from Promega (USA). The dCas9-miR-524 plasmid DNA was constructed by Viewsolid Biotech (Beijing, China).

Synthesis of PEI-PBA. The synthesis of PEI-PBA was achieved by conjugating 2-bromomethylphenylboronic acid (PBA) onto branched polyethylenimine ($PEI_{1.8K}$). Briefly, 1.80 g of PEI (Mw = 1.8 kDa) was firstly dissolved in methanol to reach a concentration of

120 mg/mL, following by the addition of 0.54 g of PBA. The reaction solution was stirred under reflux at 70 °C for 12 h, and then the product was precipitated by dropping the reaction solution into cold ether. The product was dried under vacuum to achieve pale yellow solid (yield 70%). The successful conjugation was confirmed using 1H nuclear magnetic resonance spectrum (^{1}H NMR). 1H NMR (400 MHz, D_2O, δ ppm): δ=7.4-8.0: (4H, ArH), δ=2.2-3.6: (4H, $-CH_2NHCH_2-$).

Synthesis of $mPEG_{113}$-b-$PLys_{100}$. The $mPEG_{113}$-b-$PLys_{100}$ was synthesized by the ring-opening polymerization of Lys(Z)-NCA using MeO-PEG_{113}-NH_2 (Jinpan biotech, Shanghai, China) as the initiator. Briefly, Lys(Z)-NCA (5.35 g, 18.4 mmol) was dissolved in 30 ml of DMF. The polymerization was initiated by the addition of MeO-PEG_{113}-NH_2 (0.61 g, 0.123 mmol). The reaction mixture was stirred for 3 days at 35 °C under a dry argon atmosphere. After the reaction, the solvent was evaporated under reduced pressure. The resulting product was dissolved in 15 mL of $CHCl_3$ and then precipitated into excessive diethyl ether to obtain mPEG-b-PLys(Z) (yield 85%). Deprotection of Z group in $mPEG_{113}$-b-PLys(Z) was carried out by addition of HBr (33 wt.% in HOAc, 2 mL) to the solution of $mPEG_{113}$-b-PLys(Z) (2.0 g) in 20 mL CF_3COOH for 2 h at 0 °C. After precipitating using cold diethyl ether, the product was re-dissolved in DMF and filtered through a 0.22 µm Millipore filter. The filtrate was precipitated in excessive diethyl ether to remove the residual CF_3COOH and obtain $mPEG_{113}$-b-PLys (yield 65%). $mPEG_{113}$-b-PLys was characterized using 1H NMR. As shown in Fig. S2, the degree of polymerization (DP) of Lys was estimated to be 100 by comparing the integration of the peaks of the $-OCH_2CH_2-$ protons of PEG at 3.3-3.4 ppm and the -NHCHCO- protons of PLys at 4.2-4.4 ppm. ^{1}H NMR (400 MHz, D_2O, δ ppm): δ=4.2-4.4: (1H, -NHCHCO-), δ=3.6-3.8: (4H, $-OCH_2CH_2-$), δ=3.3-3.4: (3H, H_3CO-), δ=2.8-3.1: (2H, $-CH_2CH_2CH_2CH_2NH_2$), δ=1.2-1.8: (6H, $-CH_2CH_2CH_2CH_2NH_2$).

Synthesis of $mPEG_{113}$-b-$PLys_{100}$/DMMA and $mPEG_{113}$-b-$PLys_{100}$/SA. The synthesis of $mPEG_{113}$-b-$PLyse_{100}$/DMMA was achieved by conjugating 2, 3-dimethylmaleic anhydride (DMMA) onto $mPEG_{113}$-b-$PLys_{100}$ (Fig. S1). Briefly, 100 mg of $mPEG_{113}$-b-$PLys_{100}$ was dissolved in sodium bicarbonate buffer (pH 8.5, 50 mM) to reach a concentration of 10 mg/mL, and then 211.2 mg of DMMA (five equivalents to amine groups of $mPEG_{113}$-b-$PLys_{100}$) were added. During the reaction, the pH of the solution was maintained in the range of 8.0-8.5 using 0.2 N NaOH. After the reaction, unreacted DMMA was removed by dialysis (MWCO = 3500 Da), and $mPEG_{113}$-b-$PLys_{100}$/DMMA was obtained by lyophilization.[1] The synthesis of $mPEG_{113}$-b-$PLys_{100}$/SA was similar to that of $mPEG_{113}$-b-$PLys_{100}$/DMMA by replacing DMMA with succinic anhydride (SA). The successful synthesis was confirmed using ^1H NMR analysis (Fig. S2), indicating that approximately 90% of the amine groups on $mPEG_{113}$-b-$PLys_{100}$ reacted with DMMA or SA. ^1H NMR (400 MHz, D_2O, δ ppm): δ=4.2-4.4: (1H, -NHCHCO-), δ=3.6-3.8: (4H, $-OCH_2CH_2-$), δ=3.3-3.4: (3H, H_3CO-), δ=2.8-3.1: (2H, $-CH_2CH_2CH_2CH_2NH_2$), δ=2.2-2.5: (4H, $-OCCH_2CH_2COOH$) δ=2.4-2.6: (6H, -$OCC(CH_3)C(CH_3)COOH$), δ=1.2-1.8: (6H, $-CH_2CH_2CH_2CH_2NH_2$).

Investigation on the pH-responsiveness of $mPEG_{113}$-b-$PLys_{100}$/DMMA. The pH-responsiveness of $mPEG_{113}$-b-$PLys_{100}$/DMMA was studied by monitoring change of the peak of DMMA group using 1H NMR (400 MHz, D_2O/DCl). Briefly, the $mPEG_{113}$-b-$PLys_{100}$/DMMA was dissolved in D_2O/DCl (pH=6.5) at a concentration of 1 mg/mL at 37 °C. The solution (500 μL) was immediately analyzed using ^1H NMR to access the spectra at 0 h, 0.5 h, 1 h, 1.5 h and 2 h. The shiftiness of the characteristic peak attributed to the hydrogen adjacent to amide bond/amino group (Fig. 2a) confirmed the degradation of the DMMA group of $mPEG_{113}$-b-$PLys_{100}$/DMMA in response to the acidic environment.

Preparation of MDNP and SDNP. MDNP and SDNP were prepared by mixing the solution of PEI-PBA/pDNA polyplex with $mPEG_{113}$-b-$PLys_{100}$/DMMA solution and $mPEG_{113}$-b-

$PLys_{100}$/SA solution, respectively. PEI-PBA (0.1 mL, 1.5 mg/mL in water) and dCas9-miR-524 pDNA (0.1 mL, 250 μg/mL in water) were mixed gently and incubated for 15 min to form the PEI-PBA/pDNA polyplex. To prepare MDNP, 0.1 mL of $mPEG_{113}$-b-$PLys_{100}$/DMMA solution (3 mg/mL) was added into the solution of PEI-PBA/pDNA polyplex (0.1 mL) and incubated for 15 min at room temperature. The preparation of SDNP was achieved in a similar method by employing $mPEG_{113}$-b-$PLys_{100}$/SA (0.1 mL, 3 mg/mL) instead of $mPEG_{113}$-b-$PLys_{100}$/DMMA.

Electrophoresis, DLS, and TEM analysis of MDNP and SDNP. The agarose gel retardation assay was carried out in 0.7% (w/w) agarose gel in 1×TAE buffer at a constant voltage of 120 V for 30 min. After the electrophoresis, the gel was stained with the 0.5 mg/mL ethidium bromide solution for 30 min. The plasmid DNA bands were visualized at 365 nm using a UV gel image system (SIM135A, SIMON). DNA ladder and free plasmid DNA were used as a control.

The average sizes and zeta potentials of MDNP and SDNP were determined using dynamic light scatting (DLS) measurements. The DLS measurements were performed on a laser light scattering spectrometer (BI-200SM) equipped with a digital correlator (BI-9000AT) at 636 nm at 37 °C.

The morphology of PEI-PBA/pDNA polyplex and MDNP were observed by using transmission electron microscopy (TEM, FEI Talos F200C electron microscope). For the preparation of TEM samples, PEI-PBA/pDNA polyplex and MDNP were prepared as the solutions with pH 7.4 and the concentration of pDNA at 10 μg/mL. TEM samples were prepared by drop-coating of 2 μL PEI-PBA/pDNA polyplex and MDNP onto carbon-coated copper grids (Beijing Zhongjingkeyi Technology Co., Ltd, China). Droplets of samples were contacted with the grids for 5~10 minutes, then excess amount of samples was removed. The grid was then rinsed and stained with 1% sodium uranyl acetate (5~10 μL) for 90 seconds.

Evaluation of the non-specific protein adsorption and stability of MDNP. The non-specific adsorption of MDNP was analysed using the following method. Briefly, PBS (100 μL, 10 mM), PEI-PBA/pDNA polyplex (10 μg pDNA, 100 μL), and MDNP (10 μg pDNA, 100 μL) were mixed with 100 μL of bovine serum albumin (BSA) solution (2 mg/mL) and incubated at 37 °C for 120 min. After the incubation, all the solutions were filtered and washed 5 times with PBS (10 mM) with centrifugal filtration (MWCO = 300 kDa) to remove unabsorbed BSA. The effluent liquid was collected and tuned to 1 mL, following by measuring the BSA concentration of each sample using BCA assay. The adsorption of BSA on nanoparticles were calculated according to the following equation:

$$\text{Adsorption (\%)} = \frac{\text{adsorbed BSA in the mixture}}{\text{BSA content in the initial sample}} \times 100$$

The stability of SDNP and MDNP in PBS containing 10% FBS was examined in phosphate buffered saline (PBS, pH 7.4, 0.01 M) with 10% FBS at 37 °C, and the size of nanoparticles was characterized at various incubation times using dynamic light scatting (DLS) measurements. The DLS measurements were performed on a laser light scattering spectrometer (BI-200SM) equipped with a digital correlator (BI-9000AT) at 636 nm at 37 °C.

Fluorescence resonance energy transfer (FRET) assay. The acidic responsiveness of MDNP and SDNP was investigated via FRET analysis. Briefly, PEI-PBA and mPEG$_{113}$-b-PLys$_{100}$/DMMA or mPEG$_{113}$-b-PLys$_{100}$/SA was labelled with Cy3 and Cy5 to yield Cy3-PEI-PBA, Cy5-mPEG$_{113}$-b-PLys$_{100}$/DMMA, and Cy5-mPEG$_{113}$-b-PLys$_{100}$/SA according to the manufacturer's instruction. PEI-PBA/pDNA polyplex, MDNP and SDNP were prepared using the fluorescence-labeled polymers. PEI-PBA/pDNA polyplex, MDNP and SDNP were dispersed in PBS (pH=7.4, 10 mM) with the same pDNA concentration (10 μg/mL), and then measured fluorescent emission spectra at the excitation wavelength of 515 nm. As a comparison, MDNP and SDNP were also dispersed in PBS (pH=6.5, 10 mM) with the same

pDNA concentration (10 μg/mL) and incubated for 2 h. The pH was re-adjusted to 7.4 after the incubation, and the fluorescence spectra of these solutions were collected in the same method.

Cell culture. LN-229 and MDA-MB-231 were maintained in DMEM supplemented with 10% (v/v) heat-inactivated fetal bovine serum (FBS), 100 units mL-1 penicillin, and 100 μg mL-1 streptomycin. All cell cultures were maintained in 5% CO2 humidified environment at 37 °C.

Analyses of cellular uptake and endosomal escape. Cellular uptake of MDNP was studied using confocal laser scanning microscope (CLSM, Olympus, FV1000) and flow cytometry (Guava, easyCyte 8HT). Briefly, MDA-MB-231 cells were seeded at a density of 1×10^4 cells per well in a 35 mm confocal dish (Φ =15 mm) and incubated overnight for cell attachment. The cells were then exposed to MDNP and SDNP containing 1 μg YOYO-1 labeled pDNA respectively, and then incubated in complete culture medium at different pH (pH 7.4 and pH 6.5, respectively) for 2 h. After the incubation, the cells were rinsed with ice-cold PBS and fixed with fresh 4% paraformaldehyde for 15 min at room temperature. The cells were further counterstained with DAPI for cell nucleus and rhodamine phalloidin for F-actin following the manufacturer's instructions. After the staining, the cells were observed using CLSM (Olympus, FV1000). The cellular uptake efficiency of MDNP and SDNP was also assessed using flow cytometry analysis. Briefly, MDA-MB-231 cells were seeded into 6-well plates at a density of 1×10^5 cells per well. After the overnight growth, cells were exposed to various nanoparticles containing 3 μg YOYO-1 labeled pDNA and incubated in complete culture medium at different pH (pH 7.4 and pH 6.5, respectively) for 2 h. After the trypsin digestion and centrifugation, the cells were collected, washed with cold PBS and fixed with fresh 4% paraformaldehyde for the flow cytometry analysis (Guava, easyCyte 8HT). All of these experiments were performed in triplicate.

The endosomal escape capacity of MDNP was evaluated by analyzing the colocolization of the endosomes/lysosome and pDNA after internalizing into cell. Briefly, MDA-MB-231 cells were seeded at a density of 1×10^4 cells per well in 35 mm confocal dish (Φ =15 mm) and incubated overnight for cell attachment. The cells were then incubated with MDNP containing 1 μg TOTO-3 labeled pDNA in complete culture medium at pH 6.5. At 1, 2 and 4 h post the exposure of MDNP, the cells were stained with LysoTracker Green according to the manufacture's instruction. After the endosome/lysosome staining, the cells were washed twice with ice-cold PBS and fixed with fresh 4% paraformaldehyde for 15 min at room temperature, and then counterstained with DAPI for the easy observation of the cell nucleus. All the cells were observed using a CLSM (Olympus, FV1000).

In vitro gene transfection. To evaluate the transfection efficiency of the MDNP and SDNP at different pH, the pDNA encoding tdTomato fluorescent protein and the pDNA encoding luciferase protein were employed as reporter genes for the gene transfection studies. Briefly, cells were seeded into 24-well plates at 2×104 cells per well and incubated overnight in 0.5 mL DMEM with 10% FBS (v/v). Before the transfection, the culture medium was replaced with the fresh one (containing 0 or 10% serum based on the purpose) and adjusted to either pH 7.4 or 6.8. 50 μL of MDNP, SDNP and other comparative samples were added into the cell cultures with 1 μg pDNA/well, respectively. After 4 h incubation, the culture medium was replaced with 0.5 mL fresh medium containing 10% FBS (v/v) for further 48 h incubation. PEI_{25K}/pDNA polyplex was employed as positive controls to perform the same studies. At the end of experiment, the cells were rinsed with PBS. The luciferase activity was evaluated using Luciferase Assay Kit and normalized with the amount of proteins (RLU/ mg protein) in the lysates determined by BCA assay. The tdTomato fluorescent protein expression was observed by fluorescence microscope (CX41, Olympus) and quantified by

flow cytometry (easyCyte 8HT, guava). All of these transfection studies were performed in triplicate.

Quantitative real-time PCR assay evaluating the activation of miR-524 expression *in vitro*. Quantitative real-time PCR (qRT-PCR) was used to detect the Pri-miR-524 expression activated by MDNP/dCas9-miR-524. Briefly, MDA-MB-231 and LN-229 cells were seeded into 6-well plates at a density of 2×10^5 cells/well and incubated overnight in DMEM with 10% FBS (v/v). The culture medium was replaced and adjusted to either pH 7.4 or 6.8, following by the addition of 100 μL of MDNP/dCas9-miR-524, SDNP/dCas9-miR-524, MDNP/NC, PEI25K/dCas9-miR-524, respectively (3 μg pDNA/well). After 4 h incubation, the culture medium was replaced with 2 mL fresh medium containing 10% FBS (v/v) and incubated for another 48 h. After the incubation, the total RNA was extracted from the cells using TRIzol reagent (Invitrogen, USA) according to the manufacturer's instructions. A stem-loop–specific primer (GenePharma, Shanghai, China) was used to measure the expression levels of Pri-miR-524. Expression of U6 was used as an endogenous control. The miRNA was converted to cDNA using the PrimeScript RT reagent kit (TaKaRa, Tokyo, Japan) according to the manufacturer’s protocol. cDNAs were quantified by SYBR PremixExTaq (TaKaRa, Japan) by DNA Engine Opticon 2 Two-Color Real-Time PCR Detection System (Bio-Rad, USA). Fold changes for the expression levels of Pri-miR-524 were calculated using the comparative cycle threshold (CT) method (2-ΔΔCT).

Western blotting analysis. Western bolt was employed to measure the protein expression levels of Smad2, Hes1 and Tead1. Briefly, MDA-MB-231 and LN-229 cells were seeded into 6-well plates at a density of 1×10^5 cells/well and then treated as the previous description. After the transfection, each group of cells was washed with PBS for three times and then solubilized in 1% Nonidet P-40 lysis buffer. Homogenates were clarified by centrifugation at 20000g for 15 min at 4 °C, and protein concentrations were determined with a BCA assay.

Total protein lysates were separated by SDS-PAGE on 10% SDS acrylamide gels, which was then transferred to PVDF membranes (Millipore, USA). The membranes were incubated with primary antibodies against Hes1, Tead1 and Smad2 (1:1000 dilution; Santa Cruz Biotechnology, Santa Cruz, CA, USA) overnight, followed by incubating with an HRP-conjugated secondary antibody (1:1000 dilution; Zhongshan Bio Corp, Beijing, China) for 1 h. GAPDH (1:1000 dilution; Santa Cruz Biotechnology) was set as a loading control.

In vitro cytotoxicity analysis. The cytotoxicity of PEI-PBA, $mPEG_{113}$-b-$PLys_{100}$/DMMA and $mPEG_{113}$-b-$PLys_{100}$/SA were determined using CCK-8 viability assay. Briefly, cells (MDA-MB-231 and LN-229) were seeded in 96-well plates at 5×10^3 cells per well and grown to 70-80% confluence, followed by replacing the culture medium with the fresh ones containing different polymers at varied concentrations (Fig. S3) for further 24 h incubation. CCK-8 was mixed with DMEM at a volume ratio of 1/9 (freshly prepared) to achieve the CCK-8 working solution. After the incubation, the cells were rinsed using PBS buffer, followed by the addition of 100 µL CCK-8 working solution mixture and another 2 h of incubation. Quantification of the cell viability was achieved by measuring the absorbance with Tecan's Infinite M200 microplate reader (λ= 450 nm). The cell viability was calculated by referring to that of the cells without any treatment.

The in vitro anti-tumor effect of MDNP/dCas9-miR-524 were also evaluated by CCK-8 viability assay in a similar method. Briefly, MDA-MB-231 and LN-229 cells were seeded into 96-well plates at a density of 5×10^3 cells/well and incubated overnight in DMEM with 10% FBS (v/v). The cultures were adjusted to either pH 7.4 or 6.8, and then added 10 µL of MDNP/dCas9-miR524, SDNP/dCas9-miR-524, MDNP/NC, PEI25K/dCas9-miR-524, respectively (200 ng pDNA/well). After 4 h incubation, the culture medium was refreshed and further incubated for 24 h, 48 h and 72 h. After the incubation, the cells were rinsed using PBS, and the viability was accessed using CCK-8 assay.

In vivo distribution of MDNP. To investigate on the tumor accumulating ability of MDNP, female BALB/c nude mice at 4-week old were purchased from the animal center of the Cancer Institute of Chinese Academy of Medical Science, and were bred at Compare Medicine Center, Tianjin Medical University. All experimental protocols were conducted within Tianjin Medical University guidelines for animal research and were approved by Institutional Animal Care and Use Committee. The tumor-bearing mice were established by subcutaneous injection of MDA-MB-231 cells (5×10^6 for each mouse) in the mammary fat pad. The mice were randomly divided into three groups. When the tumor volume was about 400 mm^3, the mice were intravenous injected with 100 μL of PEI25K/pDNA polyplex, MDNP and SDNP containing 10 μg TOTO-3 labeled pDNA. At 1 h, 6 h and 24 h post-injection, the mice were sacrificed, and the major organs and the tumors were collected for ex vivo evaluation. Ex vivo images were taken by IVIS Lumina imaged system (Caliper Life Sciences, USA). The fluorescence images were analyzed using Living Image 3.1 (Caliper Life Sciences). To determine TOTO-3 labeled pDNA distribution in the tumor tissues, the tumor was fixed by 4% paraformaldehyde at 4 °C for 24 h and then treated with 30% sucrose solution (w/w) at 4 °C overnight. The tumor tissues were frozenly sectioned into 8 μm slices and air-dried for 30 min. After staining the nuclei with DAPI, the slices were observed using a CLSM (Olympus, FV1000).

Immunogenicity evaluations assessed with inflammatory cytokine secretion. 12 Male KunMing mice of 8-10-week-old were divided into 2 groups and respectively injected with 100 μL of PBS and MDNP/dCas9-miR-524 containing 10 μg pDNA per mouse via tail vein. The blood samples were collected from the mice 72 h after injection and stored overnight at 4 °C in a coagulant tube, which allows the blood to coagulate naturally. The blood samples were then centrifuged at 2000 rpm for 20 minutes, and the levels of IL-6, IFN-γ, TNF-α and NF-kB in the supernatant was assessed using a mouse IL-6, IFN-γ, TNF-α and NF-kB ELISA

kits following the protocol provided by the manufacture. The measurement was performed on a Tecan's Infinite M200 microplate reader.

In vivo tumor growth inhibition by MDNP. To investigate tumor growth inhibition with MDNP/dCas9-miR-524 in vivo, the tumor-bearing mice with MDA-MB-231 xenograft was established as described above. When the tumor volume was around 25 mm^3 at 10 days after cell implantation, the mice were randomly divided into five groups (five mice per group) and intravenously injected with 100μL of PBS, MDNP/dCas9-NC, PEI_{25K}/dCas9-miR-524, SDNP/dCas9-miR-524 and MDNP/dCas9-miR-524 containing 10 μg plasmid DNA per mouse every three days. Tumor growth was monitored by measuring the perpendicular diameter of the tumor using calipers. The estimated volume was calculated according to the formula: tumor volume (mm^3) = 0.5 × length × $width^2$.

Detection of Pri-miR-524 expression in tumors and normal organs. For analysis the *in vivo* upregulation of Pri-miR-524, the mice administrated with MDNP were sacrificed, and their tumors and other organs (e.g., heart, liver, spleen, lung, kidney) were collected, freezed with liquid nitrogen and grind. The RNA was collected and analyzed by qRT-PCR as describe above.

RNA in situ hybridization detection of miR-524 in tumor tissues. In situ hybridization assay was performed on freshly frozen tissue sections. In brief, slices were washed with 1× phosphate buffered saline (PBS) containing 0.5% Triton X-100. The slices were then incubated with appropriate amount of anti-DANCR, anti-miR-524 oligodeoxy-nucleotide probes (BersinBio, Guangzhou, China) with hybridization solution containing 1% blocking solution in humid chamber at 37 °C overnight. After the incubation, the slices were washed three times for 5 min each at 42 °C with 0.1% Tween-20 in 4× sodium citrate buffer (SSC), once for 5 min in 2× SSC and once for 5 min in 1× SSC in dark. After rinsing with 1 × PBS for 5 min for three times at room temperature and staining with hematoxylin, the slices were

observed using a microscope (CX41, Olympus) for evaluating the expression level of miR-524.

Immunohistochemistry analysis and H&E staining. For immunohistochemistry (IHC) analysis and H&E staining, tumor tissues were immersed in 4% paraformaldehyde at 4 °C for 24 h, followed by incubating with 30% sucrose solution (w/w) overnight. The tissues were then embedded in OCT (optimal cutting temperature compound) before storing at -80 °C. 8 μm of tissue slices were prepared with cryosections and air dried for 30 min at 25 °C for IHC analysis. For Hes1, Tead1, and Smad2 analyzing, the fixed tumor sections were incubated with primary antibodies (1:100 dilutions) overnight at 4 °C, followed by incubating with biotin-labeled secondary antibody (1:100 dilutions) for 1 h at 37 °C. The sections were then incubated with ABC-peroxidase and diaminobenzidine (DAB), counterstained with hematoxylin, and visualized using light microscope (CX41, Olympus). For the deoxynucleotidyl transferase dUTP nick end labeling (TUNEL) apoptosis studying, the fixed tumor sections were stained using TUNEL Apoptosis Assay Kit according to the manufacturer's protocol. DAPI was used for nuclear counterstaining.

Statistical Analysis. Statistical comparisons were achieved using one-way ANOVA with Dunnett post-test with GraphPad Prism 5.0.

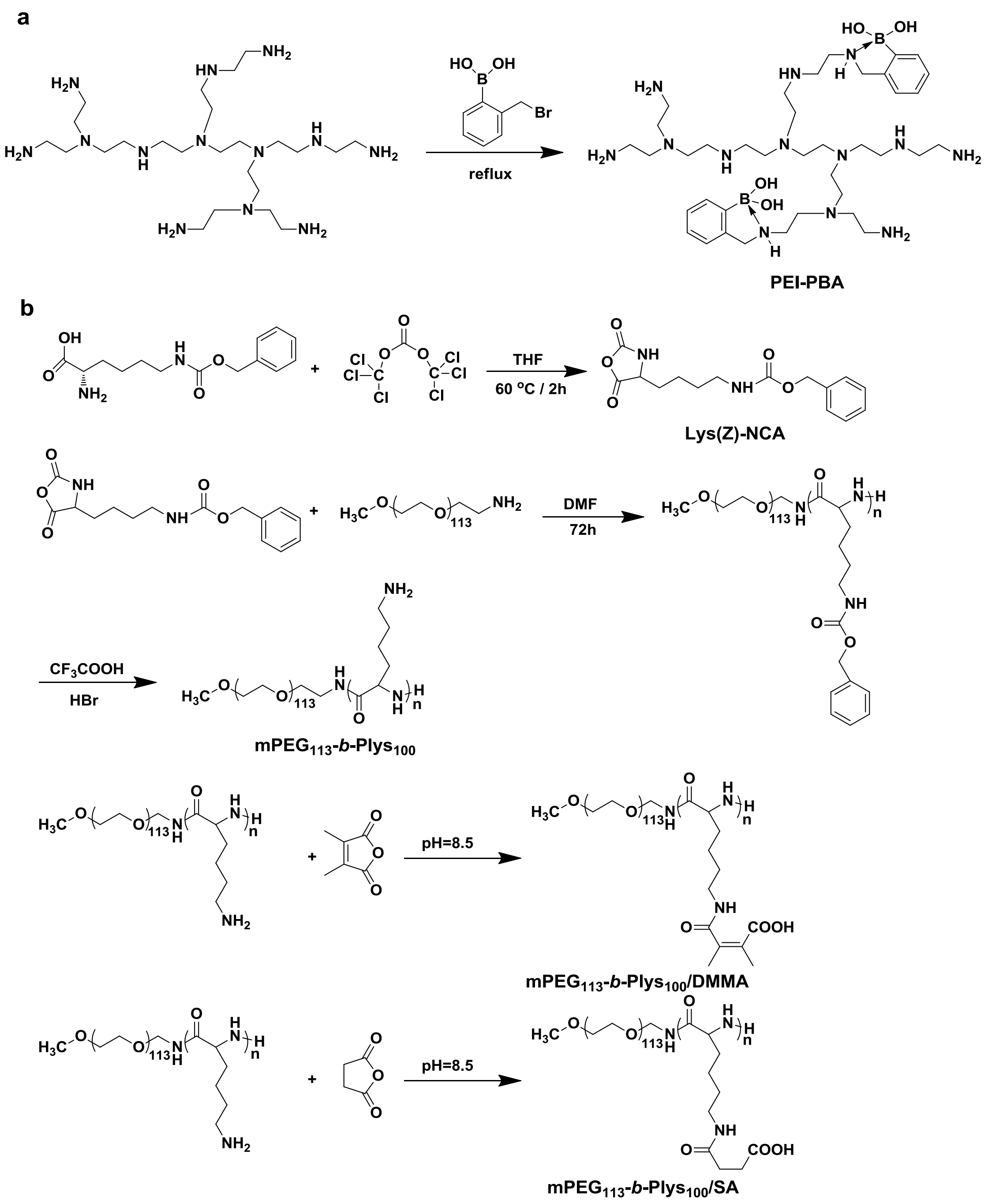

Figure S1. Synthesis routes of PEI-PBA (a), mPEG$_{113}$-*b*-PLys$_{100}$/DMMA and mPEG$_{113}$-*b*-PLys$_{100}$/SA (b).

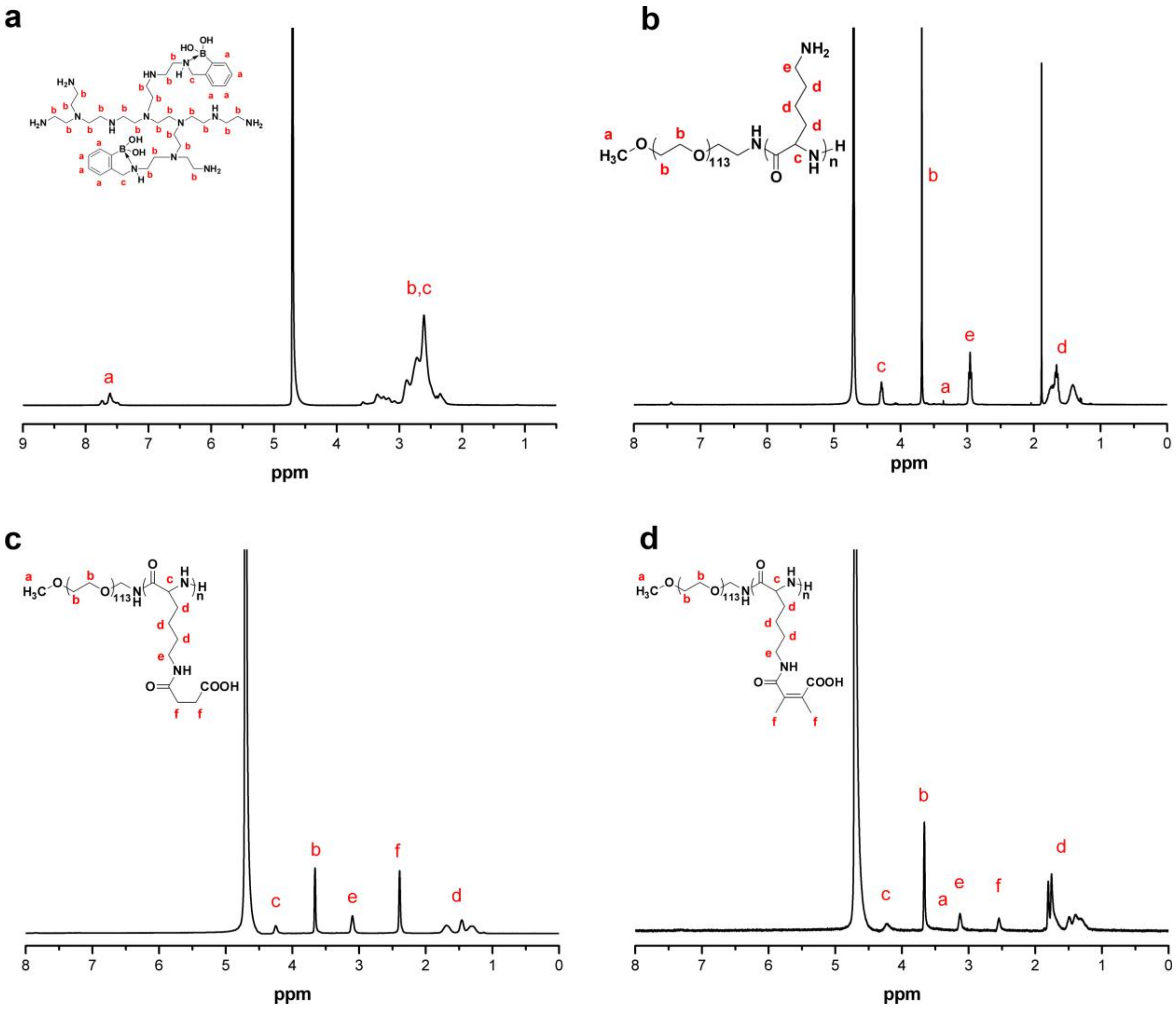

Figure S2. ^{1}H NMR spectra of PEI-PBA (a**)**, mPEG$_{113}$-*b*-PLys$_{100}$ (b**)**, mPEG$_{113}$-*b*-PLys$_{100}$/SA (c**)** and mPEG$_{113}$-*b*-PLys$_{100}$/DMMA (d**)** in D_2O.

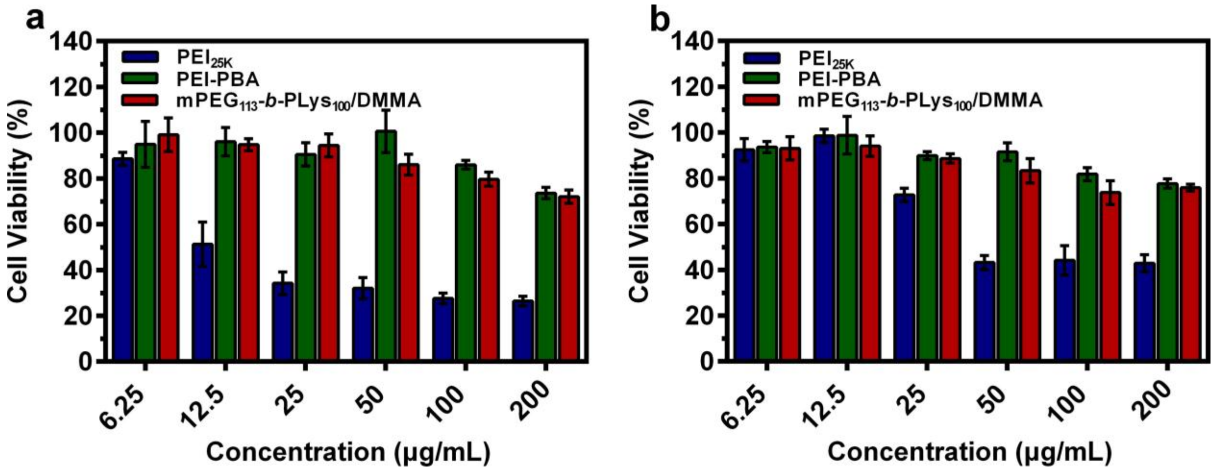

Figure S3. The cytotoxicity of PEI-PBA, mPEG$_{113}$-*b*-PLys$_{100}$/DMMA to LN-229 (a) and MDA-MB-231 cancer cells (b) at various concentrations. Data represent mean ± standard deviation (s.d.) from three independent experiments (n = 3).

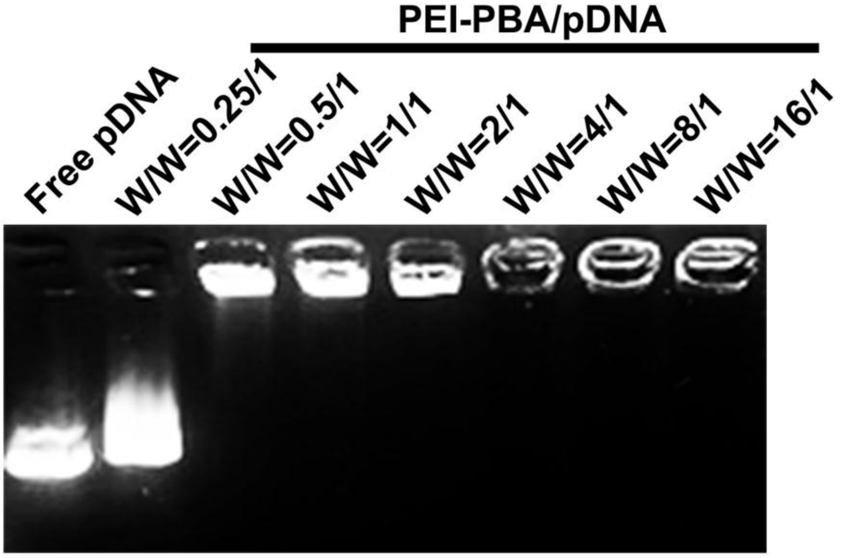

Figure S4. Gel electrophoresis presenting the capability of PEI-PBA to condense pDNA at different weight ratios. Free pDNA was used as a control.

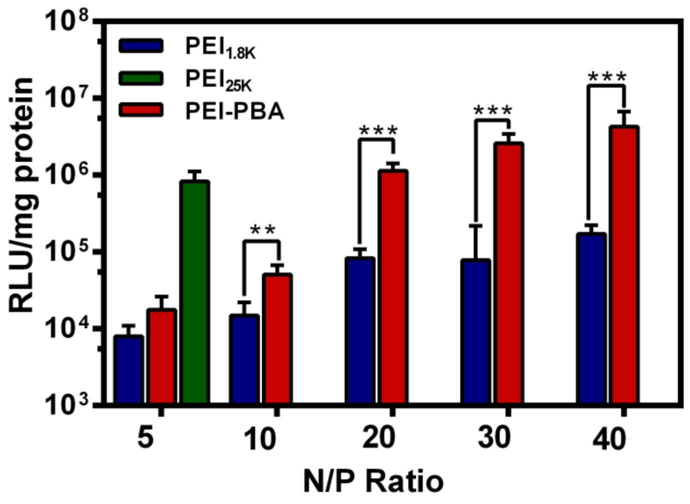

Figure S5. Luciferase expressions in LN-229 cells transfected with PEI-PBA/pDNA at different N/P ratios, PEI_{25K} and $PEI_{1.8K}$ were used as controls. Data represent mean ± s.d. from three independent experiments (n = 3) and the significance levels are **P<0.01, ***P<0.001.

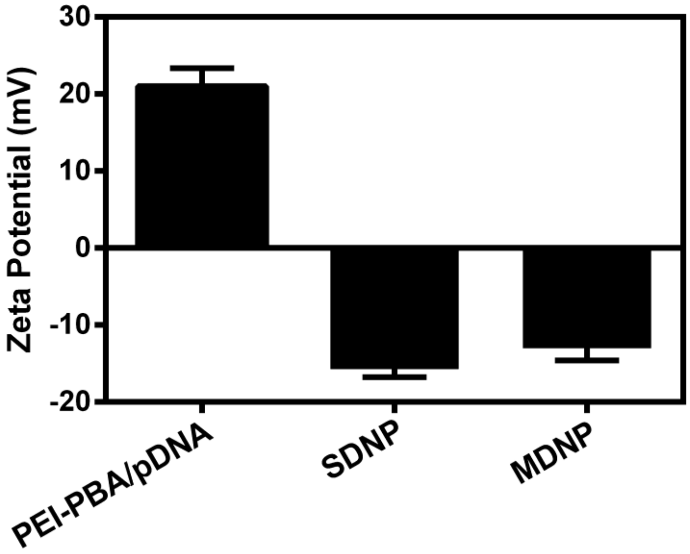

Figure S6. Zeta potentials of PEI-PBA/pDNA polyplex, SDNP and MDNP. Data represent mean ± s.d. from three independent experiments (n = 3).

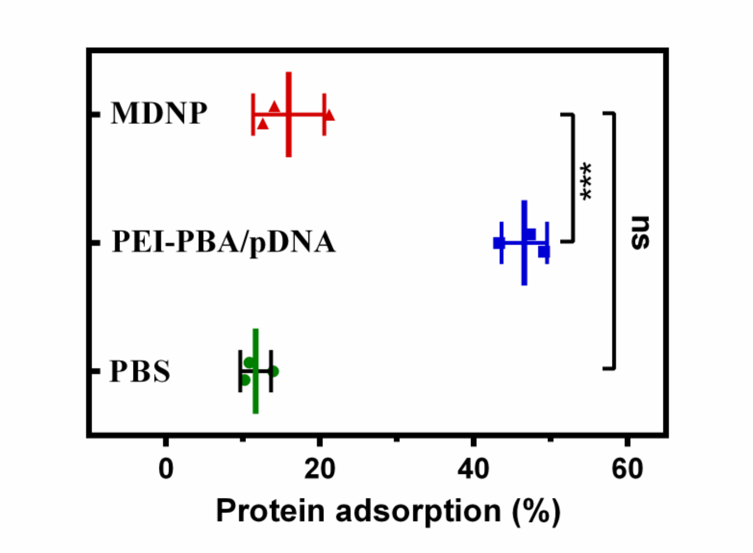

Figure S7. Quantitative measurements of BSA adsorption of PEI-PBA/pDNA polyplex and MDNP after incubation with BSA solution (1 mg/mL) for 1 h. Data represent mean ± s.d. from three independent experiments (n = 3) and the significance levels are ***P<0.001.

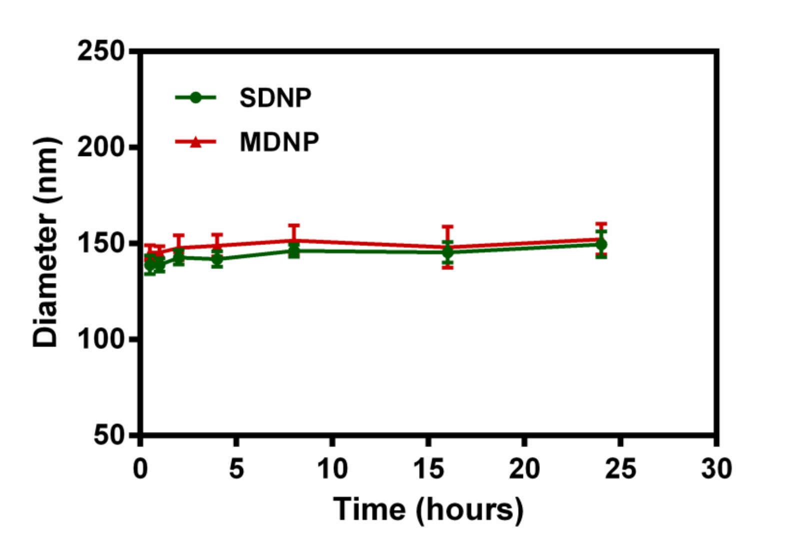

Figure S8. Stability of SDNP and MDNP in PBS containing 10% FBS.

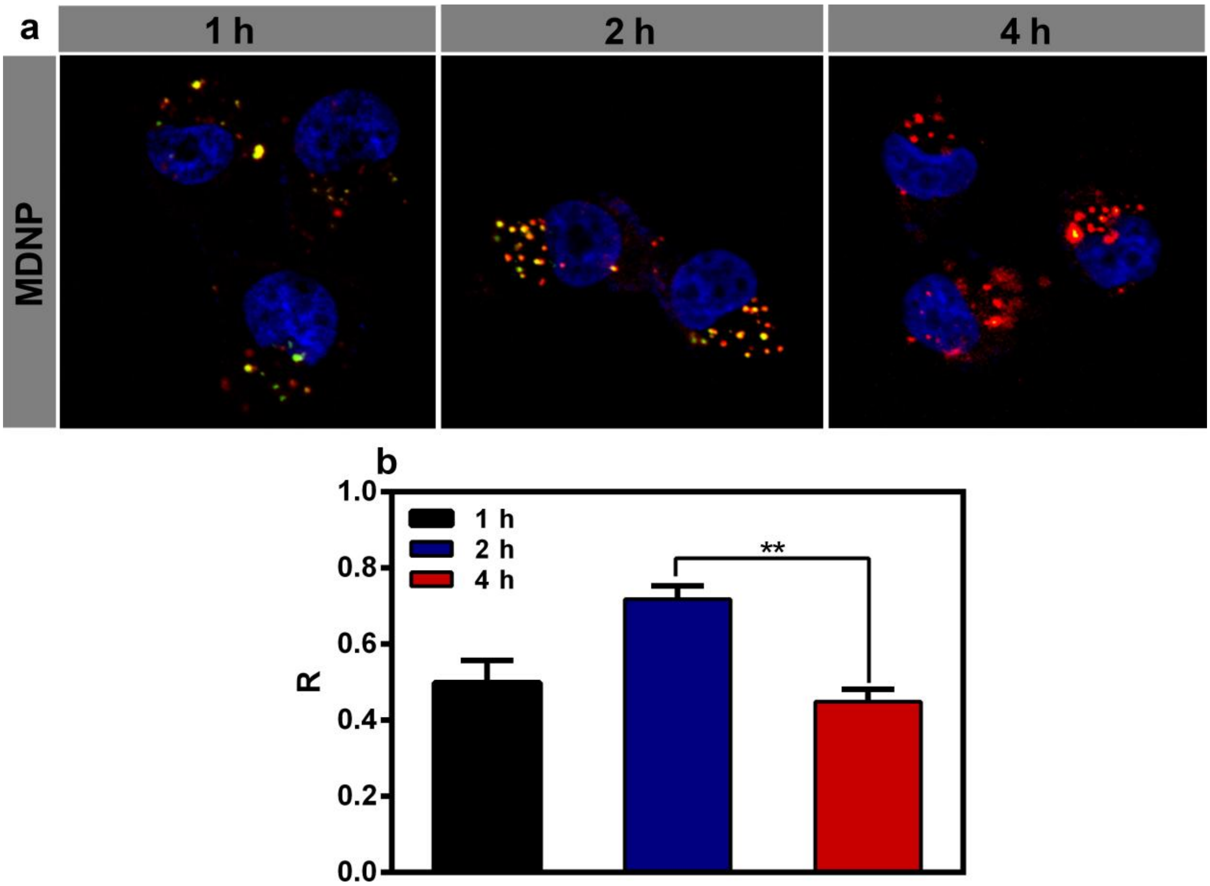

Figure S9. a) Endosomal escape of MDNP containing TOTO-3 (red) labeled pDNA. Endosomes and lysosomes were stained with LysoTracker Green, and the nuclei were stained with DAPI (blue). b) R value which represents the colocalization degree of TOTO-3 and LysoTracker Green was calculated from the CLSM images for evaluating the endosomal release efficiency. Data represent mean ± s.d. from three independent experiments (n = 3) and the significance levels are **P<0.01.

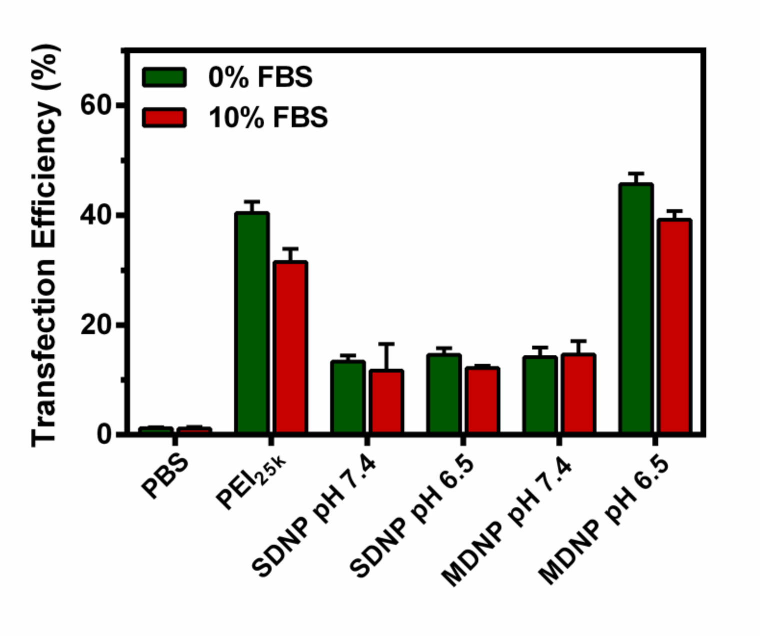

Figure S10. *In vitro* transfection efficiency of SDNP and MDNP at pH 7.4 and pH 6.5. The transfection efficiency was assessed by measuring the expression levels of tdTomato fluorescent protein in LN-229 cells using flow cytometry after the transfection. PEI_{25K} was used as the positive control. Data represent mean ± s.d. from three independent experiments (n = 3).

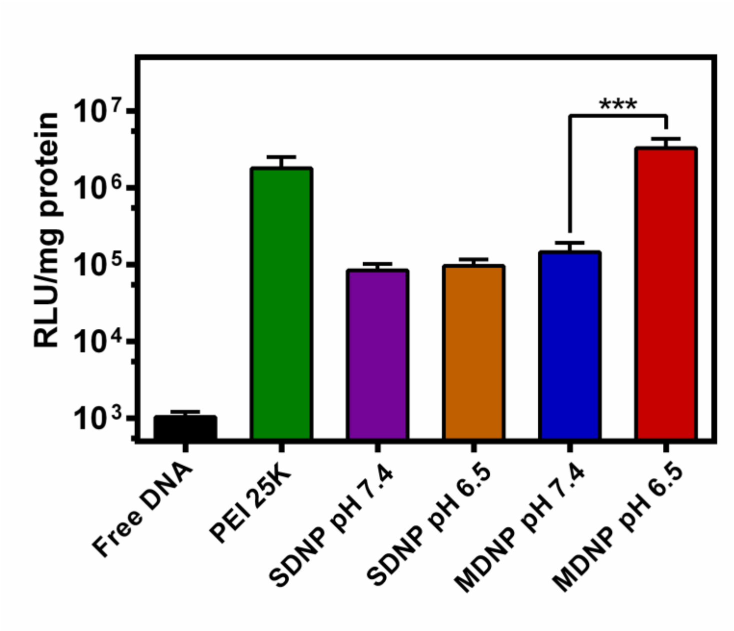

Figure S11. *In vitro* transfection efficiency of the SDNP and MDNP at pH 7.4 and 6.5. The transfection efficiency was assessed by measuring luciferase expression in LN-229 cells after the transfection. Data represent mean ± s.d. from three independent experiments (n = 3) and the significance levels are ***P<0.001.

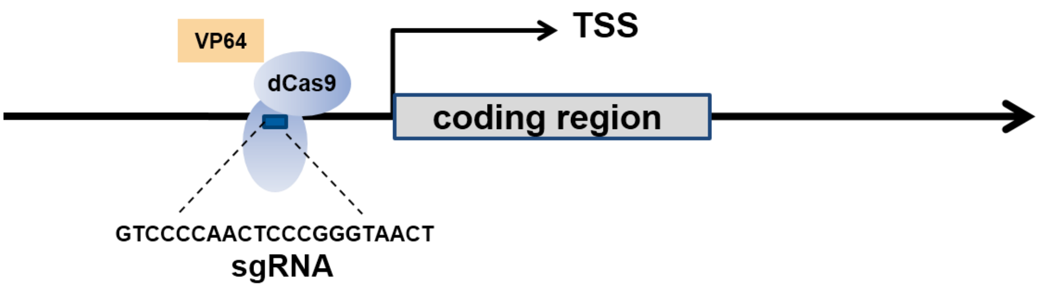

Figure S12. Schematic diagram of dCas9-miR-524 system.

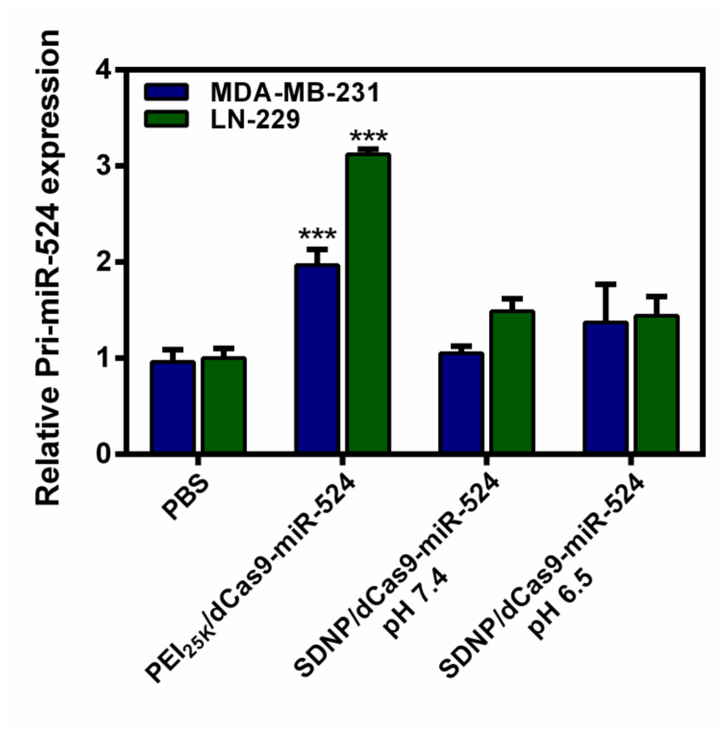

Figure S13. Relative expression level of Pri-miR-524 in LN-229 and MDA-MB-231 cells after transfecting with SDNP/dCas9-miR-524 at different pH. The expression levels of Pri-miR-524 were detected by quantitative real-time PCR assay (qRT-PCR). PEI_{25K} was employed as the positive control. Data represent mean ± s.d. from three independent experiments (n = 3) and the significance levels are ***P<0.001.

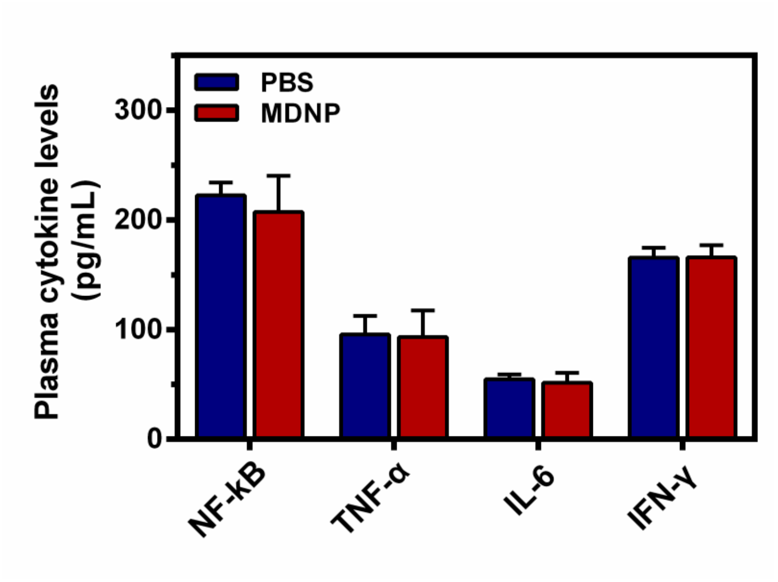

Figure S14. Plasma cytokine levels after the injection of MDNP. PBS was used as control. IFN-α, interferon α; IL-6, interleukin 6; TNF-γ, tumor necrosis factor γ; NF-kB, nuclear factor kB. Data represent mean ± s.d. from six independent experiments (n = 6).

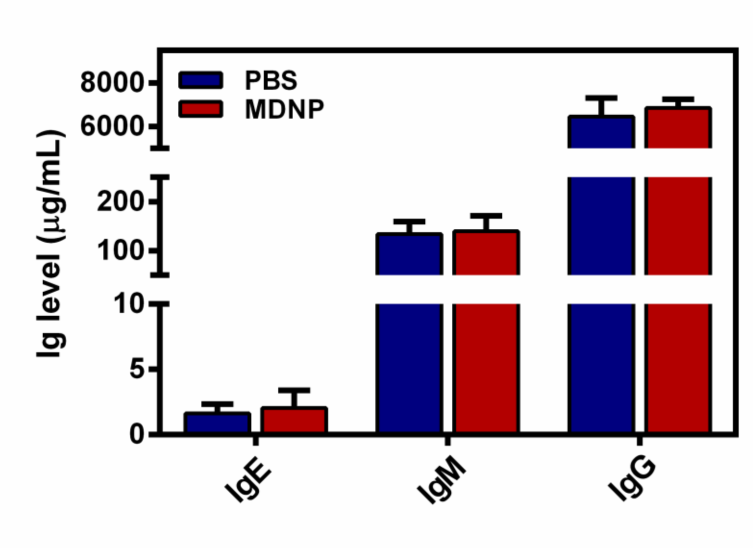

Figure S15. The levels of IgE, IgM, IgG after the injection of MDNP. PBS was used as control. Data represent mean ± s.d. from six independent experiments (n = 6).

References

[1] Y. Ding, C. Du, J. W. Qian, L. Z. Zhou, Y. Su, R. Zhang, C. M. Dong, *Polym. Chem.* **2018**, *9*, 3488.
